# *SPIRRIG* is required for BRICK1 stability and salt stress induced root hair developmental plasticity in Arabidopsis

**DOI:** 10.1007/s44154-024-00190-w

**Published:** 2024-11-25

**Authors:** Chi Zhang, Jingyu Zeng, Wenjuan Xie, Chuanseng Liu, Linyu Niu, Yanling Wang, Yali Wang, Muyang Shi, Jingxia Shao, Wenjia Wang, John Schiefelbein, Fei Yu, Lijun An

**Affiliations:** 1 State Key Laboratory of Crop Stress Resistance and High-Efficiency Production and College of Life Sciences, Northwest A&F University, 22 Xinong Rd, Yangling, Shaanxi 712100 China; 2 Department of Molecular, Cellular, and Developmental Biology, University of Michigan, Ann Arbor, MI 48109 USA; 3 CAS Center for Excellence in Molecular Plant Sciences, Chinese Academy of Sciences, Shanghai, 200032 China; 4 Chengdu Institute of Biology, Chinese Academy of Sciences, Chengdu, 610041 China

**Keywords:** Actin cytoskeleton, ARP2/3, SCAR/WAVE, Protein stability, Root hair

## Abstract

Developmental plasticity is critical for plants to adapt to constantly changing environments. Plant root hairs display dramatic plasticity under different environments and therefore play crucial roles in defense against environmental stressors. Here, we report the isolation of an Arabidopsis mutant, *salinity* *over-sensitive* *mutant 1–1* (*som1-1*), also exhibiting root hair developmental defects. Map-based cloning and allelic analyses confirmed that *som1-1* is a new mutant allele of *SPIRRIG* (*SPI*), which encodes a Beige and Chediak Higashi (BEACH) domain-containing protein. *SPI* has been reported to facilitate actin dependent root hair development by temporally and spatially regulating the expression of BRICK1 (BRK1), a subunit of the SCAR/WAVE actin nucleating promoting complex. Our living cell imaging examinations revealed that salt stress induces an altered actin organization in root hair that mimics those in the *spi* mutant, implying *SPI* may respond to salt stress induced root hair plasticity by modulating actin cytoskeleton organization. Furthermore, we found *BRK1* is also involved in root hair developmental change under salt stress, and overexpression of *BRK1* resulted in root hairs over-sensitive to salt stress as those in *spi* mutant. Moreover, based on biochemical analyses, we found BRK1 is unstable and *SPI* mediates BRK1 stability. Functional loss of *SPI* results in the accumulation of steady-state of BRK1.

**Supplementary Information:**

The online version contains supplementary material available at 10.1007/s44154-024-00190-w.

## Introduction

The sessile nature of plants often exposes them to unfavorable environmental conditions in the forms of biotic and abiotic stresses. To resist these threats, plants have evolved specific defensive strategies to contribute to their diversification and fitness to constantly changing environments (Zhu 2016; Yang and Guo 2018; Gong et al. 2020; Karlova et al. 2021; Zhao et al. 2021; Gul et al. 2022; Kohli et al. 2022; Kumar et al. 2023; Leisner et al. 2023; Xiao and Zhou 2023; Ibeas et al. 2024). Root hairs are cellular extensions of root epidermal cells found on nearly all vascular plants, and are considered to be important sensors and defense structures in plants (Salazar-Henao et al. 2016; Arif et al. 2019; Karlova et al. 2021; Kohli et al. 2022; Liu et al. 2023; Qian et al. 2024). The presence of root hairs extends the surface area of the root system, playing crucial roles in anchorage, storage, and mineral and water acquisition (Ruiz et al. 2020; Karlova et al. 2021; Kohli et al. 2022). In addition, root hairs are also beneficial for shaping soil structure, enhancing soil health and promoting microbial diversity (Koebernick et al. 2017; Robertson-Albertyn et al. 2017). Moreover, root hair traits like density and length display high developmental plasticity in response to different environments, which affects water and nutrient uptake and in turn overall plant growth (Ruiz et al. 2020; Karlova et al. 2021; Kohli et al. 2022; Ibeas et al. 2024). Therefore, elucidating the regulatory mechanisms associated with root hair development and stress response will be beneficial for deeper understanding of defense mechanisms in plants, and furthermore, for genetically improving stress resistant crops.

Root hair development occurs in four main stages including cell fate determination, initiation, elongation, and maturation (Grierson et al. 2014). Studies in Arabidopsis have shown that the root hair cell fate is specified via interactions between protein complexes of transcriptional activators including *WEREWOLF* (*WER*), *GLABRA 3* (*GL3*)/*ENHANCER OF GL3* (*EGL3*), and *TRANSPARENT TESTA GLABRA 1* (*TTG1*), and repressors including *CAPRICE* (*CPC*), *TRYPTICHON* (*TRY*), *EHANCER OF TRY AND CPC1* (*ETC1*)/*ETC2*/*ETC3*, and *GLABRA2* (*GL2*) in different cell files (Salazar-Henao et al. 2016). Following specification, root hair initiates and elongates through a cascade action of basic helix-loop-helix (bHLH) transcription factors (Masucci and Schiefelbein 1994; Yi et al. 2010; Datta et al. 2015).

Besides transcription factors, filamentous-actin (F-actin) also plays crucial roles in the establishment of cell polarity and the maintenance of elongation of root hairs (Bibikova et al. 1999; Stephan 2017; Bascom Jr. et al. 2018; Szymanski and Staiger 2018; Wang et al. 2020; Bi et al. 2022; Liu et al. 2023; Qian et al. 2024). In the process of root hair development, F-actin organization is in temporally and spatially dynamic changed. As root hair initiation, actin are fine, short, and form meshwork in bulges (Baluska et al. 2000; Chin et al. 2021). Accompanying the bulge elongated and transitioned to rapid tip growth, the base and shank of the cell consist of thick, longitudinal F-actin bundles along the growth direction, while the dome region of the cell still retains tip-focused dense actin network (Baluska et al. 2000; Chin et al. 2021). When root hair stops elongating, and gets mature, F-actin in tips are in thick cables instead of meshwork (Baluska et al. 2000; Chin et al. 2021). These phenotypic observations imply the precise regulation of actin organization during root hair development. As expected, people found that treatment Arabidopsis seedlings with the actin-disrupting compound latrunculin B (LatB) inhibits root hair elongation and unidirectional growth (Bibikova et al. 1999; Gibbon et al. 1999). Also, perturbing genes associated with F-actin biosynthesis or dynamics result in impaired root hairs with abnormal actin structures (Tominaga-Wada et al. 2011; Wang et al. 2020; Bi et al. 2022; Qian et al. 2024). The actin-related protein 2/3 (ARP2/3) complex is well-characterized actin nucleator in plants (Yanagisawa et al. 2013), playing crucial roles in a variety of eukaryotic cellular processes (Szymanski 2005; Yanagisawa et al. 2013; Chin et al. 2021). Functional loss of ARP2/3 complex subunits *DISTORTED1* (*DIS1*) and *CROOKED* (*CRK*) lead to the formation of wavy root hairs (Mathur et al. 2003a, 2003b). However, activation of ARP2/3 for efficient nucleation of F-actin requires the SCAR/WAVE complex that is comprised by Sra1/PIR121/CYFIP1, Nap1/NAP125, Abi-1/Abi-2, Brick1 (BRK1)/HSPC300, and Scar/WAVE (Davidson and Insall 2013; Yanagisawa et al. 2013). Among these, *BRK1/HSPC300* is critical for activation of ARP2/3 since *BRK1* genetically interacts with ARP2/3 components and selectively stabilizes the ARP2/3 activators SCAR1 and SCAR2 (Djakovic et al. 2006; Le et al. 2006). Knockdown of *HSPC300* in Drosophila cultured cells results in a reduction of cortical F-actin and alterations in cell morphology (Kunda et al. 2003). In plants, mutation of *BRK1* gene causes the loss of pavement cell lobe formation and the production of distorted trichomes with aberrantly localized cortical F-actin organization (Frank et al. 2003; Djakovic et al. 2006). However, whether there are factors that regulate *BRK1* activities remains unclear. Chin et al. (2021) reported that *SPIRRIG* (*SPI*) facilitates actin-dependent root hair development potentially by modulating BRK1 activities. *SPI* encodes a beige and Chediak Higashi (BEACH) domain containing protein (Saedler et al. 2009) and was initially isolated as one of a set of ‘*DISTORTED*’ genes, whose loss-of-function mutants show twisted and wavy trichomes (Hülskamp et al. 1994; Schwab et al. 2003). Further investigations demonstrate that *spi* mutants also display severer root hair developmental defects (Saedler et al. 2009; Chin et al. 2021). In particular, during the transition from initiation to tip growth in root hairs, BRK1 depletion coincides with SPI accumulation (Chin et al. 2021), implying the interaction between *BRK1* and *SPI* in the processes of root hair development, but the genetic evidence is currently lacking.

In addition to itsr roles in fundamental developmental processes, F-actin cytoskeleton is also critical for abiotic stress response and resilience either as a direct target or a signal transducer (Wang and Mao 2019; Lv et al. 2021; Wang et al. 2021a, b; Bi et al. 2022; Kumar et al. 2023; Sun et al. 2023; Qian et al. 2024). For example, F-actin undergoes cycles of depolymerization and repolymerization under hyper-salinity condition (Wang et al. 2010). During early stages of salt stress, actin bundles are induced, while F-actin polymerization disappears after high salt stress, indicating F-actin mediated stress response and defensive mechanisms. Consistent with this, genetic analyses demonstrated that actin-related and actin-binding proteins participate in stress response by modulating the dynamics of the F-actin cytoskeleton (Zhao et al. 2013). Under hyper-salinity condition, the ARP2/3 complex promotes actin assembly and modulates cytoplasmic Ca^2+^ levels and ion homoeostasis, which may confer plant salinity tolerance. Whether there are any other F-actin cytoskeleton components facilitate in stress response is still interested.

In this study, we showed that *SPI* and *BRK1* are required for salt stress induced root hair developmental plasticity in an F-actin cytoskeleton-dependent manner. Moreover, *SPI* modulates BRK1 stability during root hair development. Our work uncovers the key role of *SPI* and *BRK1* on root hair developmental response to environmental stressors. Furthermore, we also provide direct evidence for the regulation of *BRK1* by *SPI* at the post-translational level, addressing the molecular relationship between *SPI* and SCAR/WAVE-ARP2/3 complexes during cell morphogenesis.

## Results

### Isolation and analyses of the *som1-1* mutant

To explore the possible mechanisms that plants use to respond to environmental stress, we performed forward genetic screen through examining root hair developmental response to salt stress by using *gl2-3* EMS mutagenesis pool we established before (Shi et al. 2016). As a result, a recessive Arabidopsis mutant that exhibits hyper-sensitivity to salt stress was isolated, and was designated as *salinity over-sensitive mutant 1–1* (*som1-1*).

**Fig. 1:**
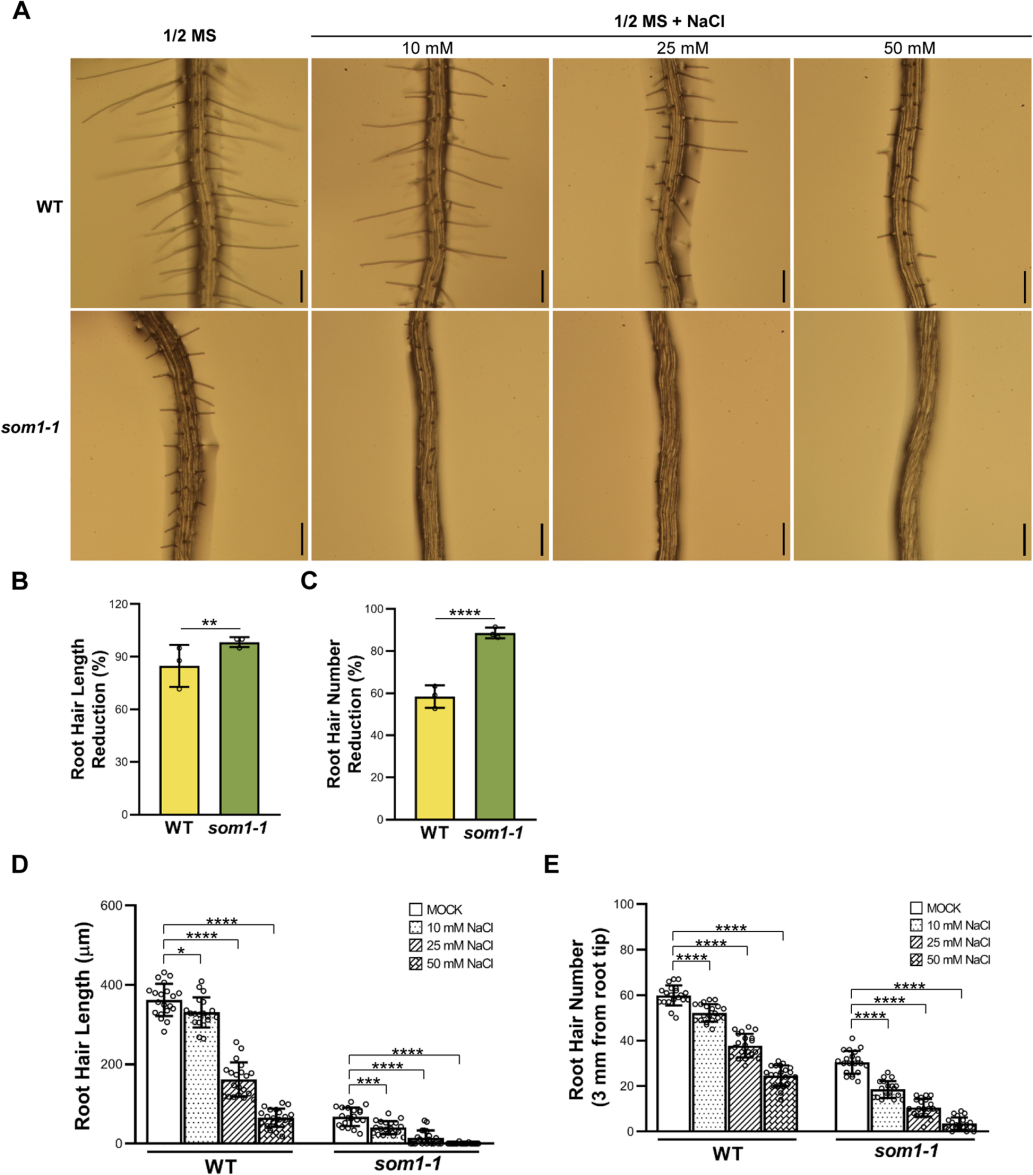
Isolation of *som1-1* mutant. **A** Root hair phenotypes of 4-day-old WT and *som1-1* seedlings grown on 1/2 MS medium with or without NaCl. Scale bars = 200 μm. **B** and **C** Reduction ration of root hair length (**B**) and root hair density (**C**) of WT and *som1-1* under 50 mM NaCl treatment. **D** and **E** Quantitative analyses of root hair length (**D**) and root hair number (**E**) in WT and *som1-1* under NaCl treatment with different concentrations. For **B** and **C**, data are mean ± SD (*n* = 20) from three biological replicates. For **D** and **E**, the experiments were repeated for three times with similar results and one set of the data was represented (*n* = 20). ****P < 0.0001, ***P < 0.001, **P < 0.01, *P < 0.05 (Student’s *t* test relative to controls)

Under our initial screen condition with 50 mM NaCl treatments, although both root hair initiation and elongation were inhibited in either the wild type (WT) or the *som1-1* plants, *som1-1* mutant showed a more dramatic response (Fig. 1A). During the treatment, the average root hair length of 4-day-old WT seedlings was decreased from ~ 362.21 ± 40.60 μm to ~ 65.11 ± 23.07 μm, with a decreased ratio of 82.82% (Fig. 1B, D). Similarly, root hair density in WT was also reduced, with a reduction ratio of 58.41% (~ 59.90 ± 4.39 before treatment and ~ 24.55 ± 4.58 after treatment, respectively) (Fig. 1C, E). In contrast, root hair initiation and elongation in *som1-1* were almost inhibited under salt stress (Fig. 1A). After 50 mM NaCl treatment, the root hair length and density were ~ 0.49 ± 1.56 μm and ~ 3.50 ± 2.74 in *som1-1* (Fig. 1D and E), with the decreased ratio of 98.35% and 88.64%, respectively (Fig. 1B and C). These results suggest that *SOM1* gene may participate in salt stress response in the aspect of plant root hair development. To confirm the participation of *SOM1* in salt stress, the *som1-1* plants were also subjected to grow in NaCl-containing medium in different concentrations and root hair phenotypes were investigated. As shown in Fig. 1A, we found a dosage-dependent response of WT and *som1-1* root hairs to salt stress. However, with the increase of the NaCl concentration, the repression of root hair elongation and initiation were significantly stronger in the *som1-1* mutant compared to the WT (Fig. 1A, D and E). To evaluate the effects of *SOM1* on the key genes that associate with root hair development, we detected the expression levels of the genes and found the transcripts levels of most of genes were reduced compared to those in the WT (Fig. S1), suggesting that *SOM1* may act upstream of these genes to regulate root hair development. Taken together, these phenotypic observations indicate the important role of *SOM1* in plant response to salt stress during root hair development.

### *som1-1* is a new mutant allele of *SPI*

**Fig. 2:**
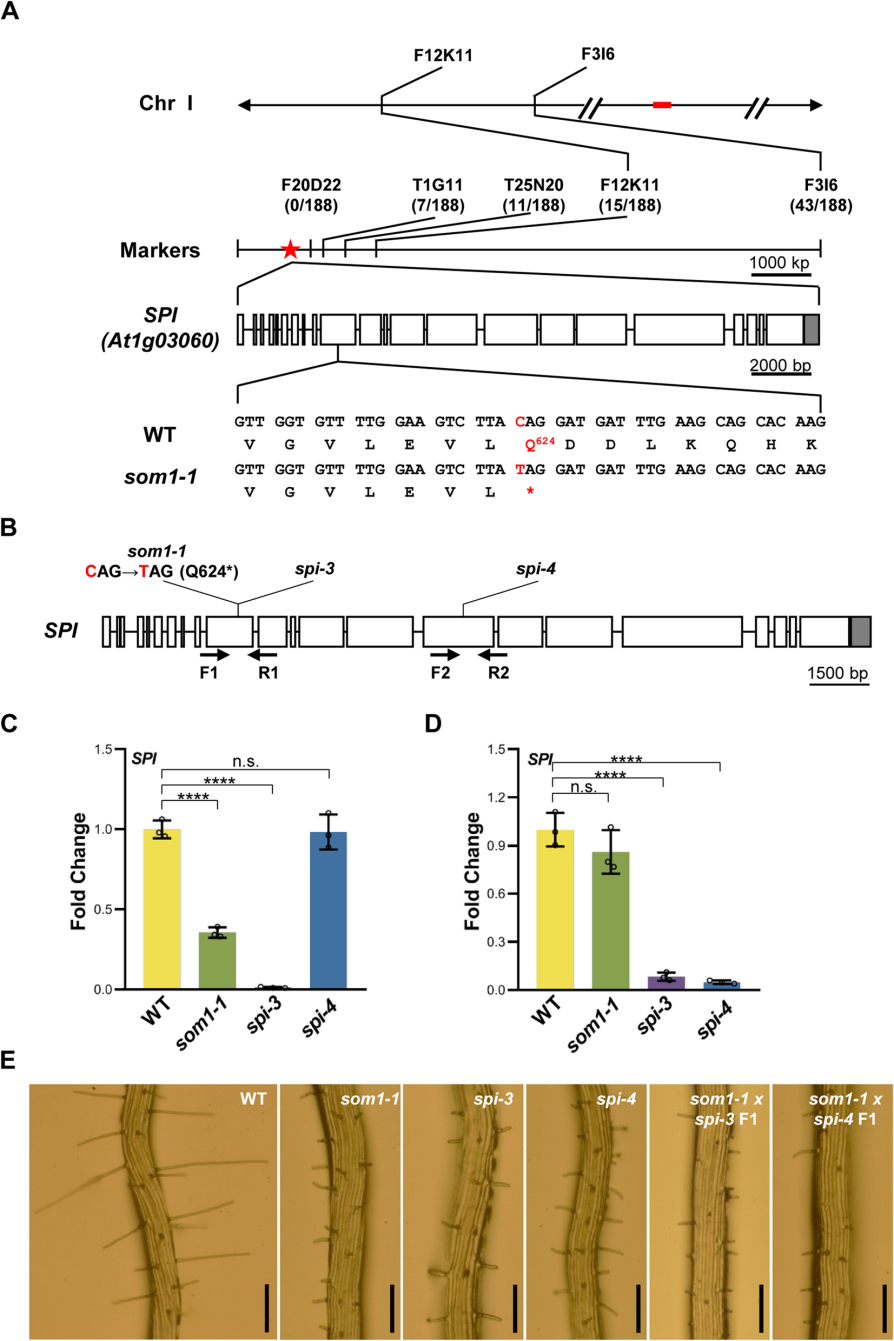
Allelic analyses of *som1-1* and *spi* mutants. **A** Map-based cloning of *SOM1* locus. *SOM1* gene was preliminarily linked to markers F12K11 and F3I6 on chromosome I through BSA analyses. Further fine-mapping mapping with additional molecular markers placed *SOM1* upstream of F20D22. Numbers of recombinants were shown under each marker. The asterisk indicated the position of *SPIRRIG* (*SPI)* gene, *AT1G03060*. In the represented gene structure, boxes and solid lines represented the exons and introns, respectively. The 5´ and 3´ untranslated regions were shown as shaded boxes. The highlighted nucleotides below the gene model indicated the exact position of the mutation site. **B** Schematic representation of the mutation sites in *som1-1*, *spi-3* (*Salk_065311*), and *spi-4* (*GK_420D09*). Boxes and solid lines represented the exons and introns, respectively. The 5´and 3´untranslated regions were shown as shaded boxes. **C** and **D** Real-time quantitative RT-PCR (RT-qPCR) analyses of transcripts levels of *SPI* in *som1-1* and *spi* alleles with primers F1 coupled with R1 in the 10th exon (**C**), and primers F2 coupled with R2 in the 15th exon (**D**). Fold changes were calculated with respect to the expression levels in the WT. Data are mean ± SD of three biological replicates. ****P < 0.0001, n.s. not significant (*P* > 0.05, Student’s *t* test). **E** Root hairs phenotypes of 4-day-old 1/2 MS medium grown WT, *som1-1*, *spi-3*, *spi-4*, *som1-1* × *spi-3* F1, and *som1-1* × *spi-4* F1 plants. Scale bars = 200 μm

To determine the genetic identity of *SOM1*, a map-based cloning strategy was employed to isolate the *SOM1* gene. Initial bulked segregant analyses (BSA) revealed that the *SOM1* locus is close to molecular markers F12K11 and F3I6 on chromosome I (Figs. 2A; S2). Fine mapping confined *SOM1* to an interval upstream of molecular marker F20D22 (Fig. 2A). Literature searches showed that the *SPI* (*AT1G03060*) gene resides in this region, and its loss-of-function mutants display similar root hair phenotypes as those of the *som1-1* mutant (Hülskamp et al. 1994; Schwab et al. 2003; Saedler et al. 2009). *SPI* encodes a 3, 571 amino acid long BEACH domain containing protein that participates in a plethora of cellular and developmental processes (Saedler et al. 2009). To determine whether an *SPI* mutation is responsible for the developmental defects of *som1-1*, a genomic fragment of *SPI* from *som1-1* was isolated and sequenced and a C to T single nucleotide transition was identified in the 10th exon of the *SPI* gene (Fig. 2A). Theoretically, this single nucleotide mutation would convert the 624th amino acid residue from glutamine to stop codon (Q624*) (Fig. 2A), resulting in a premature termination of protein translation. Due to the large size of *SPI*, we failed to generate a construct containing the native *SPI* promoter driving the full-length *SPI* cDNA or genomic DNA to carry out genetic complementation analyses. Alternatively, we obtained two independent *SPI* loss-of-function mutants *spi-3* and *spi-4* and carried out allelic tests. The *spi-3* and *spi-4* mutants carry T-DNA insertions in the 10th and 15th exons of *SPI* gene, respectively (Fig. 2B; Steffens et al. 2015). The transcripts accumulation of *SPI* in *som1-1*, *spi-3*, and *spi-4* were examined by using RT-qPCR (Fig. 2C and D). Phenotypic examination revealed that both *spi-3* and *spi-4* exhibit similar root hair as well as trichome and epidermal pavement cell defects as those in *som1-1* (Figs. 2E; S3A and B). Quantification analyses also showed similarities in decreased root hair length and root hair density, trichome branch length, and pavement cell complexity among *som1-1*, *spi-3*, and *spi-4* (Table 1; Fig. S3C). The F1 plants from crosses between *som1-1* and *spi-3* as well as between *som1-1* and *spi-4* displayed root hair, trichome, and pavement cell morphologies resembling the respective single mutants (Table 1; Figs. 2E; S2A, B and C), indicating the failure of *spi-3* and *spi-4* to complement *som1-1* in the F1 hybrids. In addition, *spi* mutants have also been reported to display salt hyper-sensitivity (Steffens et al. 2015), we found the growth of aerial parts and primary root of *som1-1* are sensitive to NaCl treatment as well (Fig. S4A-C). Collectively, these results suggest that *SOM1* represents the same genetic locus as *SPI*, and *som1-1* represents a new mutant allele of *SPI*. Thus, *som1-1* was renamed as *spi-142* based on earlier nomenclature (Saedler et al. 2009). We also investigated the effects of salt stress on *SPI* expression and found *SPI* transcript is not apparent changed (Fig. S4D), implying the effect of salinity on *SPI* may be on protein or subcellular localization level.

**Table 1:** Statistical analyses with respect to the cellular dimensions of root hairs and trichome cells in WT and *spi* mutants

**Genotypes**	**Root Hair ** **Density**	**Root Hair Length (μm)**	**Branch Length (μm)**		
			1^st^	2^nd^	3^rd^
WT	42.60 ± 6.39	175.49 ± 34.71	285.40 ± 67.96	318.90 ± 57.25	316.49 ± 79.17
*som1-1*	24.85 ± 6.31	47.40 ± 19.18	166.99 ± 44.36	368.89 ± 90.12	244.22 ± 81.95
*spi-3*	23.70 ± 5.46	33.10 ± 17.13	166.81 ± 35.73	318.14 ± 106.98	207.13 ± 65.17
*spi-4*	22.26 ± 5.23	42.19 ± 22.17	165.65 ± 68.22	346.40 ± 108.43	195.85 ± 68.70
*som1-1* x *spi-3* F1	23.90 ± 7.45	73.50 ± 28.68	190.11 ± 37.27	326.36 ± 99.46	223.08 ± 83.36
*som1-1* x *spi-4* F1	28.25 ± 4.99	73.04 ± 25.80	170.94 ± 80.04	324.55 ± 97.76	210.07 ± 83.32

Values are mean ± SD. For root hair density and root hair length analyses, 20 independent plants were used for each line. For branch length measurement, 50 trichome cells were used for each genotype

### *SPI* is required for F-actin cytoskeleton associated salt stress induced root hair development

**Fig. 3:**
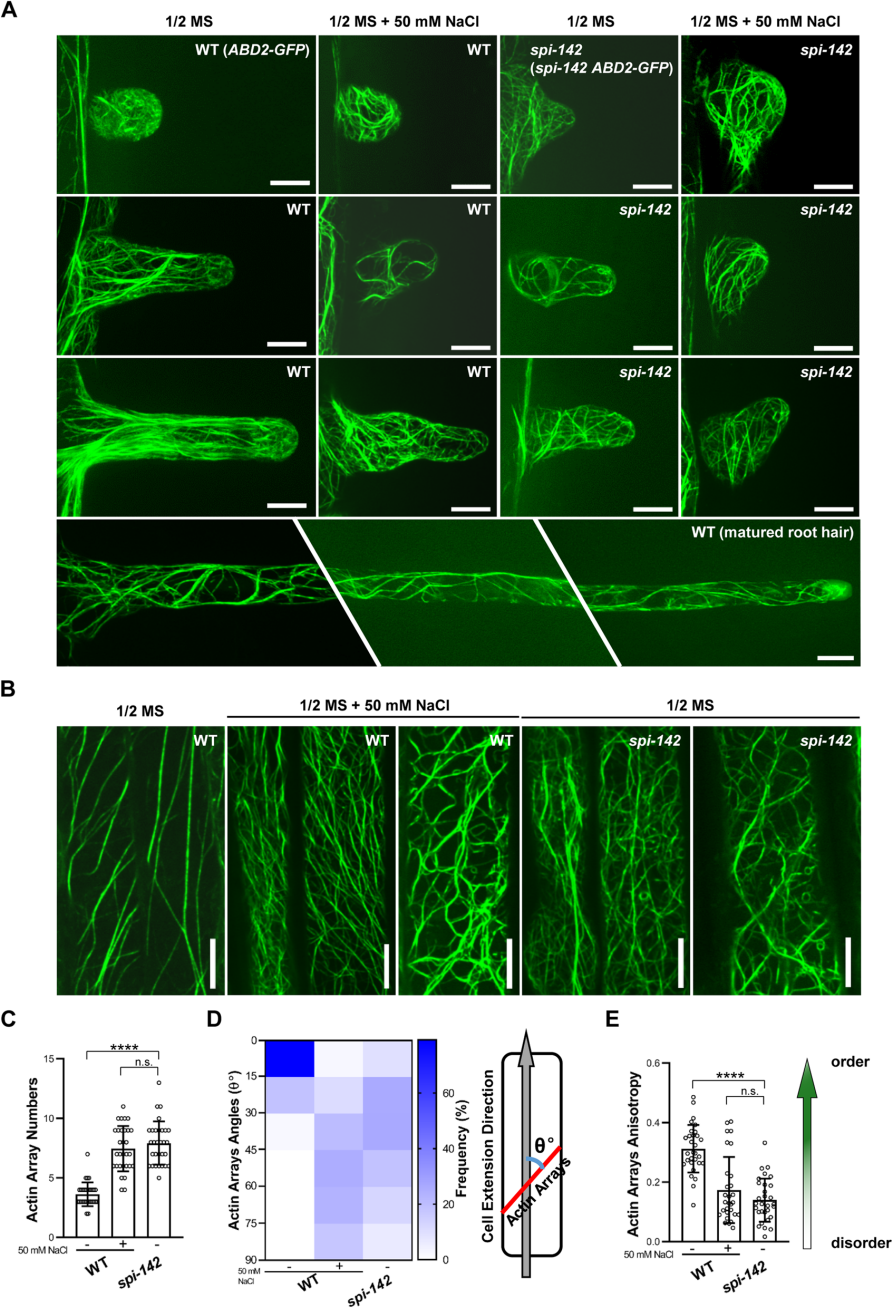
Investigation of F-actin cytoskeleton organization in WT and *spi-142* in the condition of NaCl treatment. **A** F-actin arrays in root hairs of 4-day-old *ABD2-GFP* and *spi-142 ABD2-GFP* seedlings grown on 1/2 MS with or without NaCl observed under spin-disc confocal microscopy. Scale bars = 10 μm. **B** F-actin arrays in the root epidermal cells of *ABD2-GFP* and *spi-142 ABD2-GFP* seedlings grown on medium with or without salt stress. Scale bars = 10 μm. **C-E** Quantitative analyses of the F-actin density (**C**), angles (**D**), and anisotropy (**E**) in root epidermal cells. F-actin filaments parallel to the cell’s longitudinal axis were define as 0° while those perpendicular to the cell extension direction were defined as 90°. The anisotropy score 0 was defined for nor order (purely isotropic arrays) and 1 for perfectly ordered (purely anisotropic arrays). Data are shown as mean ± SD. ****P < 0.0001, n.s. not significant (*P* > 0.05, Student’s *t* test)

Since *SPI* is demonstrated to function in actin-mediated root hair development and the actin cytoskeleton is known to play vital roles in salt stress tolerance in Arabidopsis (Wang et al. 2010; Chin et al. 2021), we asked whether the hyper-sensitivity of *spi-142* root hairs to salt stress is relevant to abnormal actin cytoskeleton organization dynamics. To this end, we examined the F-actin organization in WT (*ABD2-GFP*) and *spi-142* root hairs under salt stress by generating *spi-142 ABD2-GFP* plants. As reported before (Baluska et al. 2000; Chin et al. 2021), prior to NaCl treatment, F-actin in WT root hair bulges were in network consisting of short, discontiguous filaments (Fig. 3A), while in elongating root hairs, the base and shank parts contained thick actin bundles along the growth axis and the apical region still remained in actin meshwork (Fig. 3A). When growth stopped, thick actin bundles extended parallel or obliquely to the growth axis and also protruded to the tip of the root hairs to replace the tip-focused actin meshwork (Fig. 3A). In contrast, under NaCl treatment, distinct tip-focused F-actin meshwork was unable to form and thick F-actin bundles directly protruded to tip region in either root hair bulges or pre-matured root hairs in WT plants, which resembled those observed in untreated *spi-142* root hairs (Fig. 3A). We also examined F-actin organization in *spi-142* root hairs under salt stress, comparable phenotypes to those in the untreated *spi-142* were observed (Fig. 3A). Such an actin cytoskeleton structure may be responsible for salt stress induced formation of shorter and fewer root hairs since *spi-142* root hairs showed similar growth phenotypes (Fig. 3A). These results also suggested that the hyper-sensitivity of *spi-142* root hairs to salt stress could be due to the defects in actin cytoskeleton organization. To confirm the effects of *SPI* on salt stress induced F-actin cytoskeleton dynamics, we examined the F-actin structures in root epidermal cells as well. As shown in Fig. 3B, similar F-actin features were also observed in NaCl treated WT plants and untreated *spi-142* plants, further suggesting a certain degree of correlation between the F-actin organization and the hyper-sensitivity of *spi-142* to salt stress. As reported by Wang et al. (2010), we found prolonged salt stress resulted in F-actin depolymerization in WT plants (Fig. 3B). Consistently, measurements of F-actin density showed that WT root epidermal cells grown in 1/2 MS medium supplied with 50 mM NaCl had more F-actin arrays, which was reminiscent of those in untreated *spi-142* plants (Fig. 3C). Moreover, we also observed the formation of twisted and disorganized actin bundles induced by NaCl treatment in WT plants that was dramatically different from those regularly arranged F-actin bundles in untreated plants (Fig. 3B). Quantitative analyses of these F-actin organizations were consistent with visual observations. The angles of the actin bundles were about 0°-45°with a significant peak between 0°-15° in WT plants without salt stress, while they displayed a relatively even distribution in different orientations with a peak between 45°-60° under stress condition, like those in the untreated *spi-142* mutant (Fig. 3D). Consistently, investigation of the F-actin anisotropy exhibited that the mean anisotropy in WT without salt stress was about 0.31, compared to ~ 0.17 in WT under salt stress and ~ 0.14 in untreated *spi-142* mutant, respectively (Fig. 3E). Taken together, these results indicate that *SPI* might contribution to salt stress induced developmental response through F-actin cytoskeleton related pathways.

### *BRK1* mediates salt stress induced root hair developmental response

**Fig. 4:**
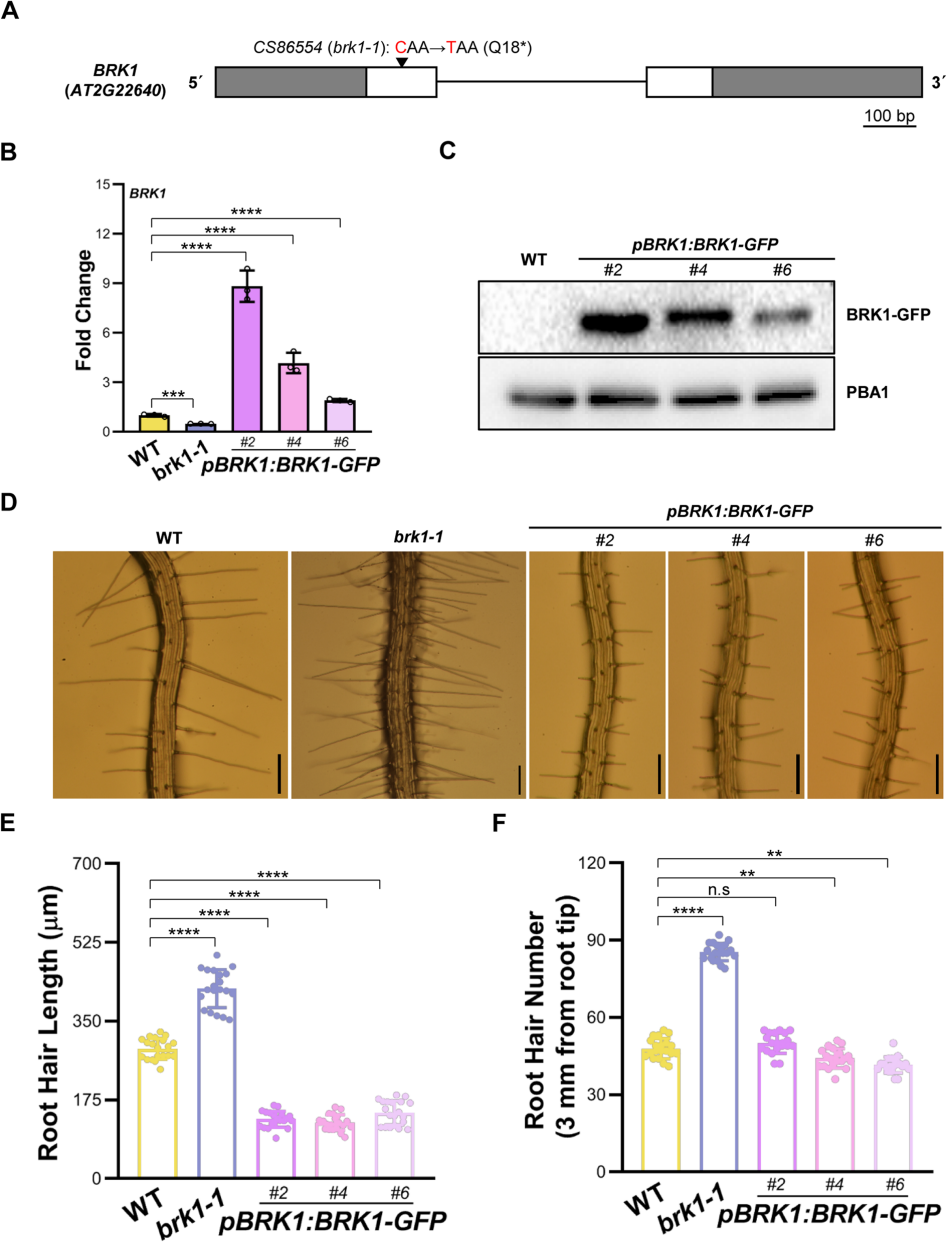
Characterization of the effects of *BRK1* on root hair development. **A** Schematic representation of the mutation site in *brk1-1* (*cs86554*). Boxes and solid lines represented the exons and introns, respectively. The 5´and 3´untranslated regions were shown as shaded boxes. **B** RT-qPCR analyses of transcripts levels of *BRK1* in WT, *brk1-1*, and independent *BRK1* overexpression lines (*pBRK1:BRK1-GFP*). **C** Immuno-blotting examination of BRK1 abundance in *BRK1* overexpression lines. The amounts of PBA1 were used as loading control. **D** Root hair phenotypes of 4-day-old 1/2 MS medium grown *brk1-1* and *BRK1* overexpression plants. Scale bars = 200 μm. **E** and **F** Root hair length (**E**) and root hair density (**F**) of WT, *brk1-1*, and *BRK1* overexpression plants. For **B**, data are mean ± SD from three biological replicates . For **E** and **F**, the experiments were repeated for three times with similar results and one set of the data was represented (n = 20). ****P < 0.0001, ** *P* < 0.01, n.s. not significant (*P* > 0.05, Student’s *t* test)

**Fig. 5:**
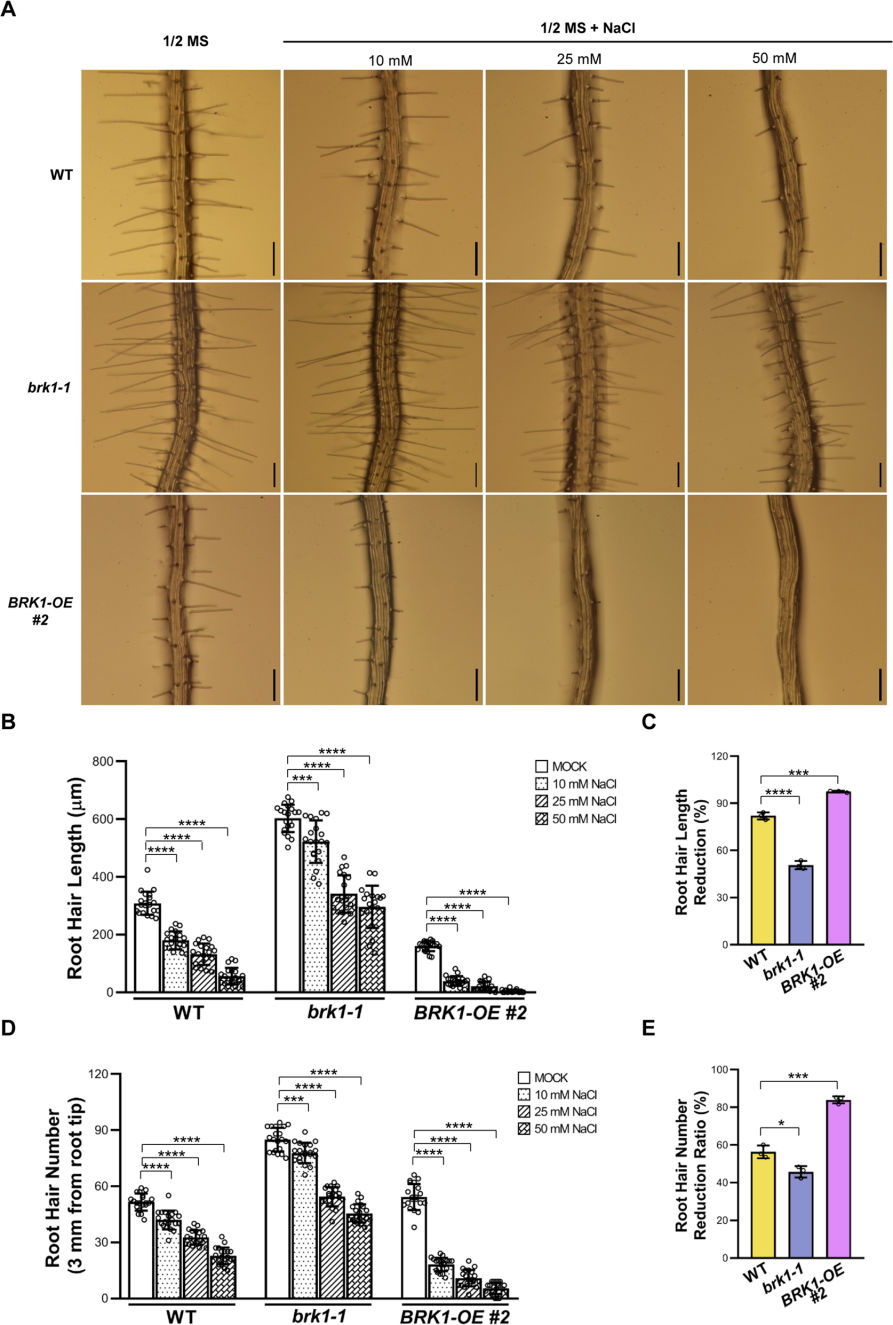
Examination of the response of *BRK1* loss-of-function mutant and overexpression plants root hairs to salt stress. **A** Root hair phenotypes of 4-day-old WT, *brk1-1, and BRK1* overexpression line (*BRK1-OE #2*) seedlings grown on 1/2 MS medium or the medium supplemented with different concentration of NaCl. Scale bars = 200 μm. **B-E** Quantification of root hair length (**B**) and root hair number (**D**) as well as the reduction ration of root hair length (**C**) and root hair number (**E**) under 50 mM NaCl treatment. For **B** and **D**, the experiments were repeated for three times with similar results and one set of the data was represented (n = 20), data are mean ± SD (n = 20). For **C** and **E**, data are mean ± SD (n = 20) from three biological replicates. ****P < 0.0001, *** *P* < 0.001, * *P* < 0.05 (Student’s *t* test)

Given that *SPI* had been shown to facilitate actin-dependent root hair development probably through temporally and spatially regulating BRK1 expression (Chin et al. 2021), and *BRK1* plays pivotal roles in F-actin nucleation (Djakovic et al. 2006; Le et al. 2006), we hypothesized whether *SPI*’s function related to F-actin in salt stress induced root hair developmental plasticity is associated with *BRK1* expression. To address this issue, we evaluated the effects of *BRK1* expression levels on root hair development under salt stress. First, we identified the *BRK1* loss-of-function mutant *brk1-1* (Fig. 4A and B; Djakovic et al. 2006), and whose root hair phenotypes were investigated. As shown in Fig. 4D, E and F, an increased root hair length and root hair density were found compared those in the WT, implying the negative regulation of *BRK1* on both root hair tip-growth and initiation. In order to confirm the influence of *BRK1* on root hair development, we also examined the root hair phenotypes of *BRK1* overexpression plants by generating *pBRK1:BRK1-GFP* transgenic plants. The *pBRK1:BRK1-GFP* construct could restore the trichome defects of *brk1-1* (Fig. S5A), indicating that *BRK1-GFP* is functional *in planta*. The overexpression of *BRK1* in *pBRK1:BRK1-GFP* plants was verified by investigating transcripts levels and protein abundance of *BRK1-GFP* through RT-qPCR and western blot analyses, respectively (Fig. 4B and C). We found in 4-day-old *pBRK1:BRK1-GFP* plants, root hair density was comparable to that of the WT (Fig. 4D, F), but root hair length was remarkably reduced, resembling to those in *spi-142* mutant (Fig. 4D and E). In WT seedlings, root hair length was ~ 288.65 ± 23.34 μm, while it was ~ 131.84 ± 17.99 μm—~ 146.14 ± 26.93 μm in *pBRK1:BRK1-GFP* plants (Fig. 4E). To test whether the reduced root hair length in *pBRK1:BRK1-GFP* is caused by reduced tip extension, we examined root hair growth rate. Like that in the *spi-142* mutant, root hair growth rate was lower in *pBRK1:BRK1-GFP* than that in the WT (Fig. S5B and C), suggesting the function of *BRK1* in root hair cell tip growth. Then, we analyzed the response of *brk1-1* and *pBRK1:BRK1-GFP* root hairs to salt stress. As shown in Fig. 5A, root hair development in *brk1-1* plants exhibited a resistance to salt stress while *pBRK1:BRK1-GFP* plants showed a hyper-sensitive response compared to those in the WT. After 50 mM NaCl treatment, root hair length and root hair density were ~ 55.86 ± 28.98 μm and ~ 22.75 ± 4.51 in the WT, whereas those were ~ 296.10 ± 73.31 μm and 45.45 ± 4.95 in *brk1-1*, and ~ 3.89 ± 4.82 μm and 5.45 ± 3.09 in *pBRK1:BRK1-GFP* plants, respectively (Fig. 5B, D). The reduction ratio of root hair length and root hair density was 82.01% and 56.35% in WT, 50.72% and 45.78% in *brk1-1*, and 97.46% and 83.99% in *pBRK1:BRK1-GFP* plants, respectively (Fig. 5C, E), which were consistent with the visual examinations. Taken together, these results indicate that *BRK1* negatively modulates root hair developmental response to salt stress and may participate in the *SPI*-mediated regulation on salt stress induced root hair developmental plasticity.

### *SPI* modulates BRK1 protein stability during root hair development

**Fig. 6:**
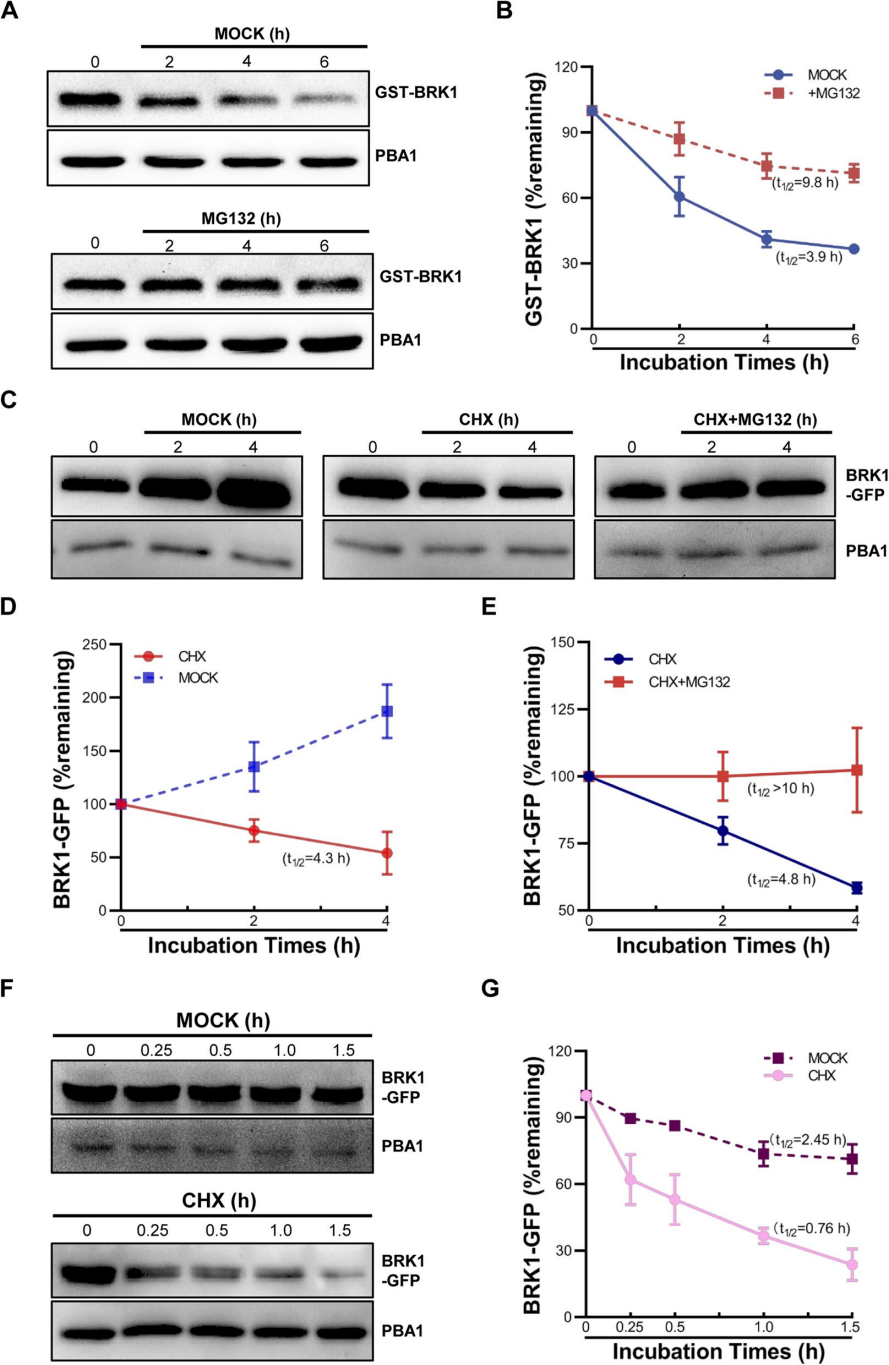
Degradation assay of BRK1. **A** In vitro cell-free protein degradation assay of recombinant protein GST-BRK1. GST-BRK1 was expressed and purified from *E. coli* and added to the cell extracts prepared from WT with our without proteasome inhibitor MG132 (200 μM). GST-BRK1 was detected with anti-GST antibody. **B** Quantitative analyses of GST-BRK1 levels in WT cell extracts with or without MG132. The half-life times were predicted based on the regression equations. **C** Protoplast-based in vivo protein degradation assay of BRK1-GFP protein. Transient expression vector *p35S:BRK1-GFP* was expressed in protoplast from WT leaf mesophyll and total proteins were extracted. The abundance of BRK1-GFP was detected when the cytosolic translation inhibitor CHX (50 μM) and MG132 were present or absent. **D** and **E** Quantification of the degradation kinetics of BRK1-GFP levels under CHX treatment (**D**) or under combined treatment with CHX and MG132 (**E**). The half-life times were predicted based on the regression equations. **F** *In planta* degradation assay of BRK1-GFP in *pBRK1:BRK1-GFP* transgenic plants. Total proteins were extracted from *pBRK1:BRK1-GFP* and treated with CHX, and BRK1-GFP levels were detected by western blot. **G** Quantitative assessments of BRK1-GFP levels in *pBRK1:BRK1-GFP* plants after CHX treatment. The half-life times were predicted based on the regression equations. For **A**, **C**, and **F**, the same volume of DMSO was used as control, and the amount of PBA1 was used as the loading control. For **B**, **D**, **E**, and **G**, data are presented as mean ± SD of three biological replicates

To investigate the possible mechanism through which *BRK1* participates in *SPI*-mediated regulation on root hair development under salt stress, we characterized whether *SPI* is involved in BRK1 stability control, given that *SPI* is required for BRK1 depletion during root hair development and the highly similar root hair phenotype of *SPI* loss-of-function mutant and *BRK1* over-expression plants (Figs. 1 and 4; Chin et al. 2021). To this end, we first investigated the stability of BRK1 through in vitro and in vivo approaches. In in vitro cell-free degradation assay, GST-BRK1 recombinant protein was expressed and purified in *E.coli* and added to total proteins extracted from 2-week-old WT rosette leaves. After monitoring the GST-BRK1 abundance during the time course, we showed that GST-BRK1 is unstable. After 6 h incubation with the cell extracts, only ~ 37% of GST-BRK1 remained (Fig. 6A and B). Quantification of the degradation of kinetics showed that the half-life (t_1/2_) was about 3.9 h. We confirmed the instability of BRK1 through a protoplast-based in vivo assay. Through transiently expressing *p35S:BRK1-GFP* in Arabidopsis leaf mesophyll protoplasts, we examined BRK1-GFP fluctuations at the indicated times. As shown in Fig. 6C and D, without cytosolic translation inhibitor cycloheximide (CHX), the amounts of BRK1-GFP continuously increased as expression times prolonged (Fig. 6C and D). However, after 4 h CHX treatment, the BRK1-GPF levels apparently decreased (Fig. 6C and D), suggesting the degradation of BRK1-GFP and instability of BRK1. In addition, we also examined *in planta* BRK1 degradation by using *pBRK1:BRK1-GFP* transgenic plants. Similar to those observed in in vitro and in vivo experiments, we found BRK1-GFP levels decreased during the time course in the presence of CHX (Fig. 6F and G), suggesting that BRK1 is unstable during plant growth.

Given that the ubiquitin/26S proteasome system (UPS) controls the turnover of numerous proteins in cells (Vierstra 2009), we analyzed whether BRK1 is subject to 26S proteasomal destruction. Recombinant GST-BRK1 protein was added to protein extracts from WT with or without the proteasome inhibitor carbobenzoxy-L-Leucyl-L-Leucyl-L-Leucinal (MG132). We found that in the absence of MG132, the amount of GST-BRK1 clearly decreased after the incubation (Fig. 6A and B). However, the abundance of GST-BRK1 was retained when MG132 was present (Fig. 6A and B), indicating the repression of BRK1 degradation and BRK1 degradation may be mediated by 26S proteasome. The 26S proteasome-dependent BRK1 destruction was further confirmed by protoplast-based in vivo assays. As shown in Fig. 6C, D, when CHX was added to the transfected cells, protein gel blotting showed a decrease of BRK1-GFP abundance within 4 h. In contrast, the BRK1-GFP levels were almost sustained when CHX and MG132 were presented simultaneously (Fig. 6C, E).

As most proteins that are subjected to UPS-dependent degradation are marked with ubiquitin chain that is covalently linked to lysine (Lys) residues (Vierstra 2009), we sought to identify the potential Lys residues that mediate BRK1 degradation. All four Lys residues in the BRK1 protein were mutated to Arginine (Arg), respectively (Fig. S6A), and the mutant proteins were expressed and purified from *E. coli*. The stability of the mutant proteins was investigated in a cell-free degradation assay. As shown in Fig. S6B and C, we found that the Lys to Arg substitution at K3 leads to apparent stabilization for GST-BRK1, suggesting that this residue may be required for ubiquitin-dependent degradation of BRK1.

**Fig. 7:**
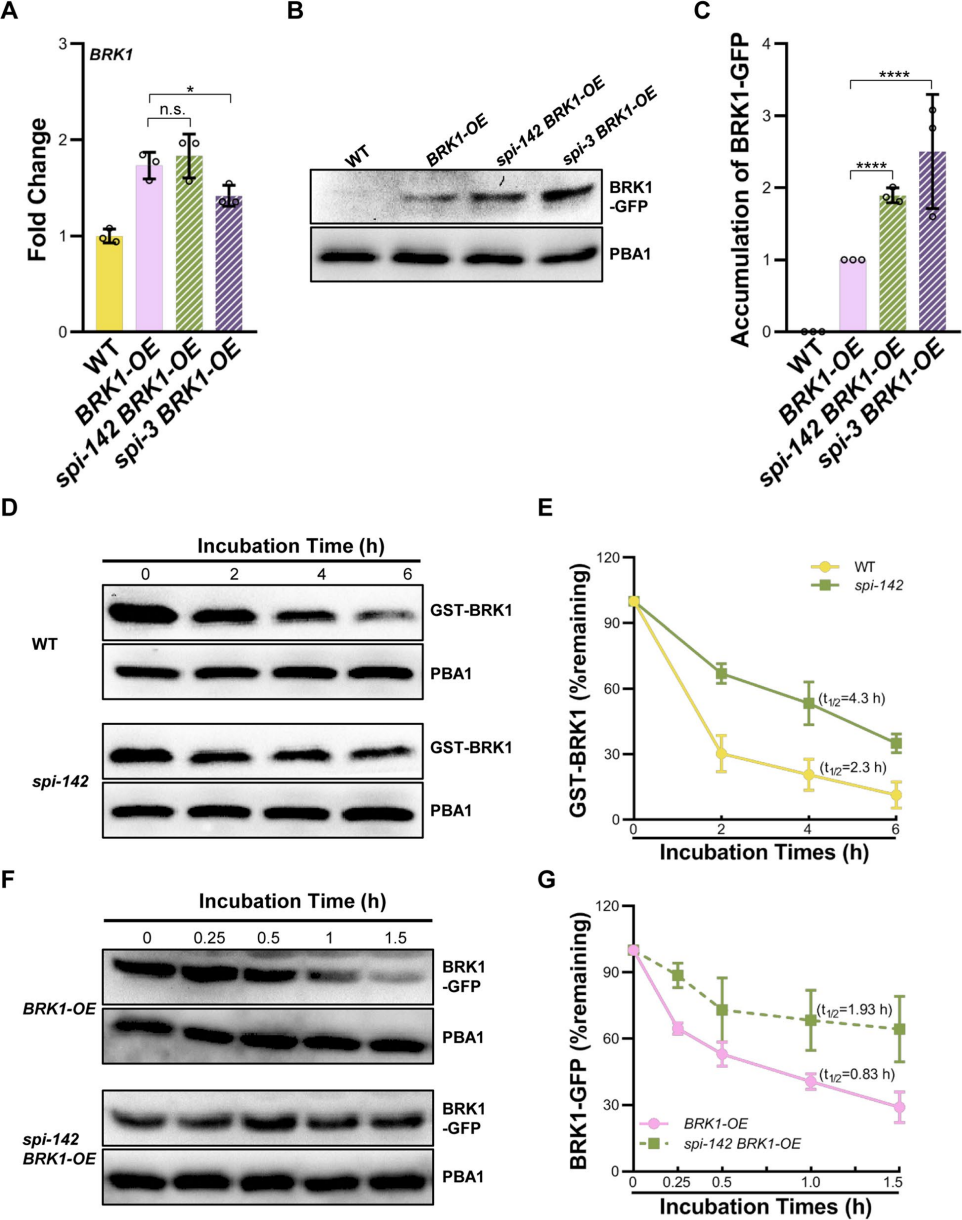
Investigation of the effects of *SPI* on BRK1 degradation. **A** Transcripts levels of *BRK1* in *pBRK1:BRK1-GFP* and *spi pBRK1:BRK1-GFP* plants examined by RT-qPCR. Fold changes were calculated with respect to the expression levels in the WT. **B** Accumulation of steady-state BRK1-GFP in WT and *spi* mutants. The amount of PBA1 was used as the loading control. **C** Quantification of the abundance of BRK1-GFP in WT and *spi* mutant backgrounds. **D** In vitro cell-free protein degradation assay of GST-BRK1 in WT and in *spi-142* mutant. Purified GST-BRK1 was incubated with the cell extracts prepared from WT and *spi-142* respectively and the abundance of GST-BRK1 was detected with anti-GST antibody. The amount of PBA1 was used as the loading control. **E** Degradation plot of GST-BRK1 in WT and in *spi-142* extracts. The half-life times were predicted based on the regression equations. **F** *In planta* protein degradation assay of BRK1-GFP protein in *pBRK1:BRK1-GFP* and *spi pBRK1:BRK1-GFP* plants. Total proteins were extracted from *pBRK1:BRK1-GFP* and *spi pBRK1:BRK1-GFP* plants and treated with CHX. BRK1-GFP levels were detected by western blot with anti-GFP antibody at the indicated times. The amount of PBA1 was used as the loading control. **G** Quantitative assessments of BRK1-GFP levels in *pBRK1:BRK1-GFP* and *spi pBRK1:BRK1-GFP* plants. The half-life times were predicted based on the regression equations. For **A**, **C**, **E**, and **G**, data are means ± SD of three biological replicates. ****P < 0.0001, *P < 0.05, n.s. not significant (*P* > 0.05, Student’s *t* test)

Next, we investigated whether *SPI* mediates BRK1 turnover by examining the effect of *SPI* inactivation on BRK1 abundance. The *pBRK1:BRK1-GFP* construct was transformed into *spi* mutants and BRK1-GFP levels were determined using anti-GFP antibodies. Transgenic lines with equivalent *BRK1* transcripts to that of *pBRK1:BRK1-GFP* were selected (Fig. 7A), and the BRK1-GFP levels were examined. As shown that in Fig. 7B and C, the steady-state levels of BRK1-GFP were higher in *spi* mutants than in the WT, suggesting the excess accumulation of BRK1 when *SPI* is inactive. To further examine the regulation of *SPI* on BRK1 protein stability, we compared BRK1 abundance in WT and in *spi* mutants using the in vitro and *in planta* degradation assays. Similar to the results shown in Fig. 7B, the amount of in vitro added GST-BRK1 decreased during the time course in the cell extracts from WT but was retained in *spi-142* cell extracts (Fig. 7D and E). After 6 h incubation, the GST-BRK1 in the cell extracts from WT was almost exhausted (~ 11% remained), while in *spi-142* cell extracts, ~ 35% GST-BRK1 protein could still be detected. These results suggested that *SPI* mutation increases the stability of BRK1. To validate the involvement of *SPI* in BRK1 stability control, we examined *in planta* degradation dynamics of BRK1 in WT and *spi* mutant background respectively. We found that when cytosolic translation is inhibited by CHX, the amount of BRK1-GFP decreased in WT after 1.5 h incubation (~ 29% remained; Fig. 7F and G). Conversely, BRK1-GFP degradation was significantly relieved in *spi* mutant background (Fig. 7F and G). After 1.5 h incubation, abundant BRK1-GFP (~ 64%) could still be detected, which was in consistent with that in in vitro experiments. Taken together, these results suggested the involvement of *SPI* in regulating BRK1 stability, and loss of *SPI* function leads to the accumulation of BRK1. In addition, we also examined the effects of NaCl treatment on BRK1 stability. As shown in Fig. S7, the amount of BRK1-GFP after treatment is higher than those before treatment, suggesting that salt stress may stabilize BRK1. Further, we found that *SPI* may involve in the influence of salinity on BRK1 stability since BRK1-GFP is more abundant in *spi* mutant than that in the WT after NaCl treatment (Fig. S7).

## Discussion

As the major communicative interface of plants and their environments, root hairs display high developmental plasticity under adverse environmental conditions and the presence of root hairs therefore serves as an effective defensive strategy for plants to cope with stress challenges (Wang and Li 2008a; Wang et al. 2020; Karlova et al. 2021; Bi et al. 2022; Kohli et al. 2022; Ibeas et al. 2024; Qian et al. 2024). Salt stress inhibits root hair initiation and tip growth in Arabidopsis (Wang et al., 2008b; Jin et al. 2023), which is considered to be an adaptive strategy of protection (Shan et al. 2005; Wang et al. 2008b; Ji et al. 2013). Root hair developmental response to salt stress was reported to be caused by ion disequilibrium and mediated by Salt Overly Sensitive (SOS) pathway (Shi and Zhu 2002, Wang et al. 2008b; Van Zelm et al. 2020). Single mutant of *SOS* genes exhibits apparent developmental-defective phenotypes for root hairs and differential salt response (Shi and Zhu 2002, Wang et al. 2008b). Recent studies showed that *SOS2* coordinates with *Rho GTPase of Plants 2* (*ROP2*) and *Rho GTPase GDP Dissociation Inhibitor 1* (*RhoGDI1*) to regulate the growth of root hair under salt stress (Liu et al. 2023). Transcription factors also serve important roles in salt stress induced root hair development. Overexpression of bHLH transcription factor ROOT HAIR DEFECTIVE 6 (RHD6) results in plants sensitive to NaCl treatment (Jin et al. 2023), while bZIP proteins ABSCISIC ACID RESPONSIVE ELEMENT-BINDNG FACTOR 1, 3, and 4 (ABF1, 3, 4) physically interact with *RHD6* and suppress its transcriptional activity to positive modulate the salinity induced inhibition of root hair development (Jin et al. 2023). Salt stress disturbs root hair growth also via affecting the biosynthesis and signaling of phytohormones (Lv et al. 2018; Wang et al. 2019). Salt stress influences the localization of auxin efflux carrier PIN-FORMED 2 (PIN2) and induced its endocytosis, as a consequences, altering auxin transport and positioning selection of root hairs (Wang et al. 2019). In crops and other plants, root hair developmental plasticity plays vital roles for plant growth as well under salt stress. *RHD3* was cloned from the salt tolerant hybrid wheat variety, and whose transcription is down-regulated under salt stress (Shan et al. 2005). Overexpression of heat shock transcription factor OsHsfA7 exhibits less, shorter root hair and salt tolerance in rice (Liu et al. 2013). *SbbHLH85* modulates resilience to salt stress by regulating root hair growth in sorghum (Song et al. 2022). These results provide theoretic evidences and candidate genes for stress molecular breeding. *Limonium bicolor* is considered as a model recretohalophyte, and can survive on high salinity environment (Yuan et al. 2022). Molecular studies showed that heterologous overexpression of bHLH transcription factor and importin-β protein SUPER SENSITIVE TO ABA AND DROUGHT 2 (SAD2) from *L.bicolor* reduces root hair number and enhances salt tolerance while expression *LbTRY* could induce root hair formation and leads plants to sensitive to salt stress (Wang et al. 2021a, b; Leng et al. 2021; Xu et al. 2021), presenting new insight into plant salt tolerance mechanisms. In this study, we report that BEACH domain containing protein *SPI* involves in salt stress induced root hair developmental plasticity in Arabidopsis. Previous studies showed that *SPI*’s function is important for root hair development as *spi* mutants exhibit shorter root hairs (Saedler et al. 2009; Chin et al. 2021). We found root hair initiation is also affected in *spi* mutants (Table 1; Figs. 1 and 2) and the growth of *spi* root hairs is hyper-sensitive to salt stress (Fig. 1), which is consistent with previous reports that *SPI* mutation leads to the growth of primary root and seedling sensitive to salinity (Steffens et al. 2015). Moreover, the developmental response of *spi* mutants under salt stress is similar to those in the *sos* mutants (Shi and Zhu 2002; Wang et al. 2008a,b), implying that *SPI* may also associate with SOS pathway to response salt environment, but the details need further investigations.

F-actin cytoskeleton dynamic plays essential roles in orchestrating root hair cell shape during development and in environmental stress response and resilience (Wang et al. 2011; Soda et al. 2016; Stephan 2017; Bascom Jr. et al. 2018; Szymanski and Staiger 2018; Wang and Mao 2019; Liu et al. 2021; Bi et al. 2022; Kumar et al. 2023; Sun et al. 2023; Liu et al. 2023; Qian et al. 2024). However, whether these two F-actin related processes work in concert to help plants to adapt and survive in unfavorable environments remains unclear. Short-term salt stress induces actin cytoskeleton assembly and bundling, but treatment with either long-term or high concentration induces actin cytoskeleton disassembly, and the SOS pathway and calcium signaling are associated with these processes (Wang et al. 2010, 2019). Actin reorganization in *sos* mutants is abnormal in response to salt stress (Wang et al. 2010). Consistently, disruption of the actin filaments with actin-filament-disrupting drugs increases death of *sos2* seedling under salt treatment conditions (Wang et al. 2010). Salt stress induces calcium accumulation in the cytosol, and ARP2/3 complex mediates calcium elevation under salinity (Qian et al. 2019; Zhao et al. 2013). Mitochondria-dependent [Ca^2+^]_cyt_ increase is enhanced in *arp2* mutant, resulting in its hyper-sensitivity to salt, suggesting an interaction between actin cytoskeleton and calcium oscillation under salinity. The actindepolymerizing factor (ADF) is highly conserved among eukaryotes and plays critical roles in the various processes of plant growth and stress response via remodeling actin cytoskeleton architecture (Sun et al. 2023). It has been showed that *ADF1* is regulated by MYB transcription factor MYB73 and is involved in response to salt stress by affecting actin filament organization (Wang et al. 2021a, b). *adf1* mutants show significantly reduced survival rate, increased percentage of actin cable, and reduced density of actin filaments under salt stress. Systematically Genome-wide identification of ADFs under various abiotic stresses in soybean (*Glycine max*) revealed that 18 *GmADF* genes show distinct expression patterns under drought and salt stresses, which may facilitate the engineering of salt-tolerant crops (Sun et al. 2023). Villin (VLN) that is considered to be one of the most important actin-binding proteins plays essential roles in plant development and in resisting adverse environments (Lv et al. 2021). *GhVLN3* and *GhVLN10* are highly and preferentially expressed in elongation cotton fibers and distinctly upregulated by abiotic stresses (Lv et al. 2021). Arabidopsis *ADF7* and *VLN1* are positively and negatively involved in root hair formation respectively (Bi et al. 2022). *ADF7* inhibits the expression of *VLN1* and leads to the decline of F-actin bundling and thick bundle formation as well as the increase of F-actin depolymerization to promote root hair formation (Bi et al. 2022). Further investigation showed that GL2 directly binds to the promoter of *VLN1* and positively regulates *VLN1* expression and actin dynamics in root hairs (Wang et al. 2020).

*SPI* has been reported to facilitate root hair development through an F-actin cytoskeleton-dependent pathway (Saelder et al. 2009; Chin et al. 2021). Moreover, *SPI* regulates the expression of genes that are relevant to salt stress response (Steffens et al. 2015), implying that *SPI* could be an integrator that responses to F-actin dependent developmental and environmental signals during root hair development, but direct evidences are currently lacking. Based on our salt sensitive experiments and the investigation of actin cytoskeleton organization, we found that *spi* mutant root hair displays similar actin structures to those in the WT under salt stress (Fig. 3), suggesting that *SPI* may mediate F-actin associated root hair developmental response under salt stress. Furthermore, based on genetic and biochemical studies, we showed *SPI* interacts with *BRK1* to modulate salt stress induced root hair developmental plasticity in F-actin cytoskeleton associated manner.

The formation of diverse actin organization is mediated by actin-binding proteins that nucleate, destabilize and bundle actin filaments (Szymanski and Staiger 2018). *BRK1* that encodes a subunit of SCAR/WAVE complex is considered to be essential for the proper organization of actin cytoskeleton because the function of actin nucleators ARP2/3 complex apparently depends on the activity of *BRK1* (Le et al. 2006; Djakovic et al. 2006). Accordingly, *BRK1* has been showed to participate in a range of cellular and developmental events and exhibit distinct expression patterns (Djakovic et al. 2006; Le et al. 2006; Liu et al. 2024). During trichome development, BRK1 is tip localized to trichome branches (Yanagisawa et al. 2018), while in the processes of root hair development, BRK1 is only expressed in the root hair initiation stage, and disappears during root hair elongation (Chin et al. 2021). This kind of specific expression pattern suggested that the activities of *BRK1* may be tightly regulated, but the pathways that associate with modulating *BRK1* activities stay to be identified. In addition, whether *BRK1* is involved in stress induced F-actin organization is also very attractive. Based on genetic analyses and NaCl treatment experiments, we found *BRK1* is relevant to salt stress and negatively regulates salt stress induced root hair development (Figs. 4 and 5). Furthermore, we also found that BRK1 is unstable and is subjected to 26S proteasome for proteolysis (Fig. 6). More importantly, we revealed that *SPI* is required for BRK1 degradation (Fig. 7). *SPI* mutation results in the accumulation of steady-state BRK1 in vitro and in vivo, which is consistent with the cellular observation by Chin et al. (2021). Our genetic analyses also showed that *BRK1* overexpression results in root hair developmental defects that resemble those in the *spi* mutants (Figs. 2E and 4D-F), further support the regulatory role of *SPI* on BRK1 stability. Considering the spatial and temporal expression differences between SPI and BRK1 during root hair development, the modulation of *SPI* on BRK1 stability may be indirectly. Studies by Steffens et al. (2015) showed that *SPI* regulates pleiotropic transcriptional changes under salt stress, so the modulation of *SPI* on BRK1 stability may be due to the transcriptional alternation of the factors that involve in ubiquitin-26S proteasome pathway, but the precise associated components need to be isolated. Taken together, our results uncover the functions of *SPI* and *BRK1* and their regulatory relationship in response to root hair development and salt stress, revealing a pathway that modulates the activities of actin cytoskeleton related protein during plant cell development and under environmental stress.

## Materials and methods

### Plant materials and growth condition

All plant materials used in the study are in the Columbia-0 (*Col-0*) background except those used for map-base cloning. The wide type (WT) refers to *Col-0* plants. T-DNA insertion lines for *SPI* (*Salk_065311*, *spi-3*; *GK_420D09*, *spi-4*) and *BRK1* (*CS86554*, *brk1-1*) were obtained from the Arabidopsis Biological Resource Center (ABRC). The T-DNA insertion sites and homozygous plants were confirmed by genomic PCR and phenotypic examination. Primer pairs used for genotyping are listed in Table S1. The marker line *ABD2-GFP* that indicates the F-actin microfilaments was kindly provided by Prof. Zhaosheng Kong (Chinese Academy of Sciences).

For plant culture, seeds were stratified at 4 °C for 2 days and then sown on commercial soil mix (Pindstrup) for gemination and growth in growth room at 22 ± 1 °C under continuous illumination (~ 100 μmol m^–2^ s^–1^). For protoplast preparation, seeds were sowed and grown on Jiffy-7-Peat Pellets (Jiffy Group) in a growth chamber (Conviron A1000) with day/night cycle (12 h/12 h) at 22 ± 1 °C, and fully expanded rosette leaves of 30-day-old plants were collected.

### Root hair, trichome, and cotyledon pavement cell phenotype characterization

Root hairs in the primary root of 4-day-old 1/2 MS-medium grown seedlings with or without NaCl were examined under the Leica M295 dissection microscope, and those in the root tips (approximately 3 mm long) were imaged using the equipped Nikon N995 digital camera. The length of the 20 longest root hairs with observable ends were measured with Image J software for each seedling (Masucci and Schiefelbein 1994). For root hair number, all visible root hairs in the root tips were counted. For measurement of the root hair growth rate, a time-lapse strategy was used as described by Szymanski and Nielsen (2009). The time interval between each measurement was 2 min, and root hair elongation was measured using Image J software.

Trichome phenotypes were examined as described by Saedler et al. (2009). Plants in different genetic backgrounds were grown in soil for 2 weeks and trichome morphologies in the 5th and 6th rosette leaves were examined with a stereoscope (Olympus, SZ61). Representative trichomes were imaged by using a tabletop scanning electron microscope (Hitachi, SEM TM3030). Trichome branch length was measured with the Image J software.

Epidermal pavement cell shapes in cotyledons of 4-day-old 1/2 MS-medium grown seedlings were examined by confocal microscopy. The outlines of pavement cells were indicated by staining with Propidium Iodide (PI) (1 mg/mL). The complexity of pavement cells was quantified using the formula: perimeter^2^/(4 × π x area) (Saedler et al. 2009).

For statistical purposes, at least 20 individual plants for each genotype in different conditions were used for analyses, and all experiments were repeated three times. Student’s *t*test was used to assess the difference.

### Map-based cloning

The *som1-1* mutant was isolated from a *gl2-3* (*Salk_039825*) ethyl methanesulfonate (EMS)-mutagenesis population we established previously (Shi et al. 2016). To identify the mutation site in *som1-1*, map-based cloning was employed according to Lukowitz et al. (2000). First, the *som1-1* mutant was crossed with Arabidopsis ecotype Landsberg *erecta* (L*er*) to generate an F2 mapping population. Then, bulked segregant analyses (BSA) were conducted with 25 pairs of molecular markers covering all 5 Arabidopsis chromosomes and a mixed genomic DNA pool with 94 individuals from the F2 segregation population. Next, fine mapping was carried out to narrow down the physical interval harboring the mutation site with additional molecular markers. Finally, candidate genes in the resulting chromosomal region were sequenced to identify the mutation site. Sequence information of the molecular markers used is listed in the Table S1.

### Protein expression and purification

To express and purify the recombinant native GST-BRK1 and mutant GST-BRK1 proteins in vitro, *pGEX4T-1-BRK1*, *pGEX4T-1-BRK1-M1* (K3R), *pGEX4T-1-BRK1-M2* (K49R, K51R), and *pGEX4T-1-BRK1-M3* (K58R) plasmids were constructed and transformed into *Escherichia coli* Rosetta (DE3) strain. The cells were cultured in liquid LB medium at 37 °C at 220 rpm until OD_600_ = 0.6. Then, GST-BRK1 recombinant proteins were induced to express with 1 mM isopropyl-ß-D-thiogalactopyranoside (IPTG). Soluble proteins were purified with Glutathione Sepharose 4B beads (17–0756-01, GE Healthcare) according to the manufacturer’s instructions. Primers used for plasmid construction are listed in Table S1.

### In vitro and in vivo protein degradation assay

The experiments were performed according to Wang et al. (2009). For in vitro degradation assay, total proteins from 2-week-old plants were extracted with degradation buffer (25 mM Tris (pH = 7.5), 10 mM NaCl, 10 mM MgCl_2_, 4 mM PMSF, 5 mM DTT, 10 mM ATP). Protein concentration was determined by the Bio-Rad protein assay, and the concentration in different plant samples were adjusted to be equal using degradation buffer. Then, about 500 ng GST-BRK1 recombinant protein was added to the total protein extracts and incubated at 22 °C for the indicated times. At each time interval, 20 μL of the reaction products were taken and boiled in the same volume SDS sample buffer for 10 min to stop the reaction. The abundance of GST-BRK1 was determined by western blot assay with anti-GST antibody (ab18184, Abcam, England).

For protoplast-based in vivo protein degradation assay, mesophyll protoplasts were isolated from the WT and transfected with transient expression plasmid *p35S:BRK1-GFP* according to Yoo et al. (2007). The transfected cells were cultured in the dark for 10 h in W5 buffer (154 mM NaCl, 125 mM CaCl_2_, 5 mM KCl, 2 mM MES). Then, the cells were treated with 50 μM cytosolic translation inhibitor cycloheximide (CHX, 66–81-9, Merk) for the indicated times. The same volume of DMSO was used as the control. Total protein was extracted from the treated cells and BRK1-GFP levels were examined by western blot with anti-GFP antibody (ab290, Abcam, England).

To perform *in planta* degradation assay, the stable expression plasmid *pBRK1:BRK1-GFP* was constructed and transformed into WT, *spi* mutants, and *brk1-1* mutant respectively. The transgenic plants were obtained through antibiotic screening, genomic PCR, and GFP florescence examination. In the T3 generation, total protein were extracted from the latest emerging rosette leaves of 2-week-old soil grown plants with degradation buffer, and treated with CHX for indicated times. The amount of BRK1-GFP protein was evaluated by western blot.

For all immuno-blotting assay, the amounts of PBA1 detected with anti-PBA1 antibody (ab98861, Abcam, England) were used as loading controls. Primers used for *p35S:BRK1-GFP* and *pBRK1:BRK1-GFP* plasmids construction are listed in Table S1.

### RNA isolation and real-time quantitative RT-PCR (RT-qPCR)

Total RNAs were extracted from the roots of 4-day-old seedlings with TRIzol reagent (15,596–026, Invitrogen, USA). The first-strand DNAs were synthesized with oligo (dT_15_) primer by using Uelris RT mix with DNase (All-in-one) (R2020, US Everbright® Inc, USA) with 1 μg total RNAs. Real-time qPCRs were carried out with the FastStart Essential DNA Green Master kit (06402712001, Roche, Switzerland) and Bio-Rad CFX96 real-time PCR system. Relative expression levels of the target genes were calculated with 2^−△Ct^. Expressions levels of *GADPH* were used as the internal controls. Primers used are listed in Table S1.

### Salt sensitivity assay

The experiments were carried out according to Wang et al. (2008b) and Jin et al. (2023). Seeds of the plants from different genotypes were surface sterilized and sown on 1/2 MS medium with or without different concentration of NaCl. Growth status of root hair, primary root, and germination of 4-day-old seedlings were examined. Root hair length, root hair density, primary root length, and biomass were quantitatively measured.

### F-actin microfilaments imaging and quantification

For *in planta* F-actin observation, the *ABD2-GFP* marker line was crossed into the *spi-142* background and unsegregated *spi-142 ABD2-GFP* plants were selected in subsequent generations by genomic PCR and GFP fluorescence analyses. F-actin organization in root hairs and root epidermal cells were examined with a spinning disk confocal system built on a DMi8 inverted microscope (Lecia) equipped with a CSU-W1 confocal scanner unit (Yokogawa) and an iXon Ultra 888 EMCCD camera (Andor) with a HC PL APO 63 × N.A.1.30 glycerol objective (Leica). Images were taken through Z-stacking with a 0.5 um z-step. GFP was excited at 488 nm.

Actin density measurements were performed according to Guan et al. (2021). Images acquired were opened with Fiji-Image J, and a line of fixed length (10 μm) perpendicular to the directions of most F-actin was draw and the numbers of F-actin across the line were counted. F-actin angles and anisotropy quantifications were performed according to Boudaoud et al. (2014). Images were loaded in Image J and angles of individual F-actin were measured. Actin filaments parallel to the cell extension direction were defined as 0° while those perpendicular to the cell’s longitudinal axis were defined as 90°. For F-actin anisotropy evaluation, the following convention was used: anisotropy score 0 = no order (purely isotropic arrays) and 1 = perfectly ordered (purely anisotropic arrays). For each scenario, a total of 8-10 seedling roots were analyzed, and 2-3 root epidermal cells with clear F-actin configuration in each seedling were selected for quantitative analyses. For F-actin angles analyses, 10 actin filaments or bundles in each cell were selected for measurements, therefore, about 300 (10 x 3 x 10) actin filaments were surveyed in each condition. Values of F-actin angle were divided into 6 intervals (0-15°, 15-30°, 30-45°, 45-60°, 60-75°,75-90°), respectively. The frequency of the F-actin angles in each interval was analyzed, and finally exhibited as a heatmap.

### Statistical analyses

Statistical analyses were carried out using Graphpad Prism software. Data are presented as means and standard diviations of the distributions. Student’s *t* test were performed to determine the significance of differences between data sets. All tests were two tailed, performed at the significance level *P* = 0.05. For all analyses, *P* < 0.05 was considered statistically significant (*P < 0.05; ** P < 0.01; *** P < 0.001; **** P < 0.0001; n.s. not significant, *P* > 0.05).

## Supplementary Information

Supplementary Material 1: Figure S1. Examination of expression levels of classical root hair genes in *som1-1* mutant. Real-time quantitative RT-PCR (RT-qPCR) was used to evaluate the transcript levels of the target genes. Fold changes were calculated with respect to the expression levels in the WT. Data are mean ± SD of three biological replicates. ****P < 0.0001, n.s. not significant (*P* > 0.05, Student’s *t* test).

Supplementary Material 2: Figure S2. Bulked segregant analyses (BSA) of *SOM1*. DNA pool were constructed by mixing the genomic DNA of 94 individuals from the F2 mapping population. *SOM1* locus was localized near F12K11 and F3I6 on Chromosome I with 25 pairs of molecular markers that evenly distributes on the five chromosomes of Arabidopsis. Green lines represented the chromosomes and red bars indicated the centromeres.

Supplementary Material 3: Figure S3. Trichome branching and cotyledon pavement cell shapes in *spi* alleles. **A** Represented trichomes in the 5th rosette leaves of 2-week-old soil grown WT, *spi* mutants, and *som1-1 × spi-3 *F1as well as* som1-1 × spi-4 *F1 plants. Scale bars = 30 μm. **B** Cotyledon pavement cells of 4-day-old 1/2 MS medium grown plants indicated in **A**. Represented pavement cells were highlighted in red. Scale bars = 30 μm. **C** Quantification of complexity of cotyledon pavement cells in different genotypes show in **A**. Data are shown as mean ± SD (n = 50).

Supplementary Material 4: Figure S4. Investigation of growth response of aerial parts and primary root of *som1-1* to salt stress. **A** The grow status of 4-day-old WT and *som1-1* seedling under salt stress. Scale bars = 2 cm. **B **and** C** Primary root length (**B**) and biomass (**C**) of 4-day-old WT and *som1-1* seedling under NaCl treatment, respectively. **D** Transcription levels of *SPI* in various salt stress condition evaluated by RT-qPCR. For **B**, data are mean ± SD (n=20); for **C **and** D**, data are presented as mean ± SD of three biological replicates. **** P<0.0001, ** *P*<0.01, n.s. not significant (*P* > 0.05, Student’s*t* test).

Supplementary Material 5: Figure S5. Complemented analyses of *brk1-1*. **A** Trichome morphology in *brk1-1* complemented lines. *In planta* expression of *BRK1-GFP* could restore the trichome defects of *brk1-1*. Scale bars = 1 cm. **B** Root hair growth curve of WT, *BRK1* overexpression plants, and *spi-142* mutant during a 20 min growth period. Scale bars = 25 μm. **C** Growth rate of the WT, *BRK1* overexpression plants, and *spi-142* root hairs. Data are presented as mean ± SD of three biological replicates. **** *P*<0.0001 (Student’s *t* test).

Supplementary Material 6: Figure S6. Identification of lysine residues mediating BRK1 stability. **A **Schematic representation of the positions of mutated lysine residues in BRK1 protein. **B **In vitro cell-free protein degradation assay of mutated recombinant GST-BRK1s. Purified mutated recombinant proteins were added to cell extracts from WT plants and protein levels were examined at the indicated time by western blot. The amount of PBA1 was used as the loading control. **C** Half-life plots for cell-free degradation of mutated recombinant GST-BRK1s. The half-life times were predicted based on the regression equations. Data are means ± SD of three biological replicates.

Supplementary Material 7: Figure S7. Dissection the effects of salt stress on BRK1 stability. **A** Western blot analyses of BRK1-GFP abundance in 4-day-old *pBRK1:BRK1-GFP*, *spi-142 pBRK1:BRK1-GFP*, and *spi-3 pBRK1:BRK1-GFP* seedlings before and after NaCl treatment respectively with antibody anti-GFP. PBA1 levels were used as the loading control. **B **Quantitative analyses of BRK1-GFP abundance after NaCl treatment in plants with different genetic background. Data are means ± SD of three biological replicates. * *P*<0.05, n.s. not significant (*P* > 0.05, Student’s *t* test).

Supplementary Material 8: Table S1. The primer pairs used in this work.

## Data Availability

All data generated or analyzed in this study are presented in this published article and its supplementary data files.

## Abbreviations

ABF1, 3, 4: Abscisic acid responsive element-binding factor 1, 3, and 4
ADF: Actin depolymerizing factor
ARP2/3: Actin-related protein 2/3
BEACH: Beige and chediak higashi
bHLH: Basic helix-loop-helix
BSA: Bulked segregant analyses
CHX: Cycloheximide
EMS: Ethyl methanesulfonate
F-actin: Filamentous-actin
GST: Glutathione-s-transferase
IPTG: Isopropyl-ß-D-thiogalactopyranoside
LatB: Latrunculin B
MG132: Carbobenzoxy-L-Leucyl-L-Leucyl-L-Leucinal
PIN2: Pin-formed 2
PMSF: Phenylmethanesulfonyl fluoride
RHD6: Root hair defective 6
RhoGDI1: Rho GTPase GDP dissociation inhibitor 1
ROP2: Rho GTPase of plants 2
RT-qPCR: Real-time quantitative RT-PCR
SAD2: Super sensitive to ABA and drought 2
SOS: Salt overly sensitive
UPS: Ubiquitin/26S proteasome system
SCAR/WAVE: Suppressor of cAMP receptor (SCAR)-WASP family verprolin homologous (WAVE) complex
