# Supplementary figures and images for: SPIRRIG is required for BRICK1 stability and salt stress induced root hair developmental plasticity in Arabidopsis
Source: Stress Biol. 2024 Nov 25;4(1):48. doi: 10.1007/s44154-024-00190-w (PMC11589064; doi:10.1007/s44154-024-00190-w)

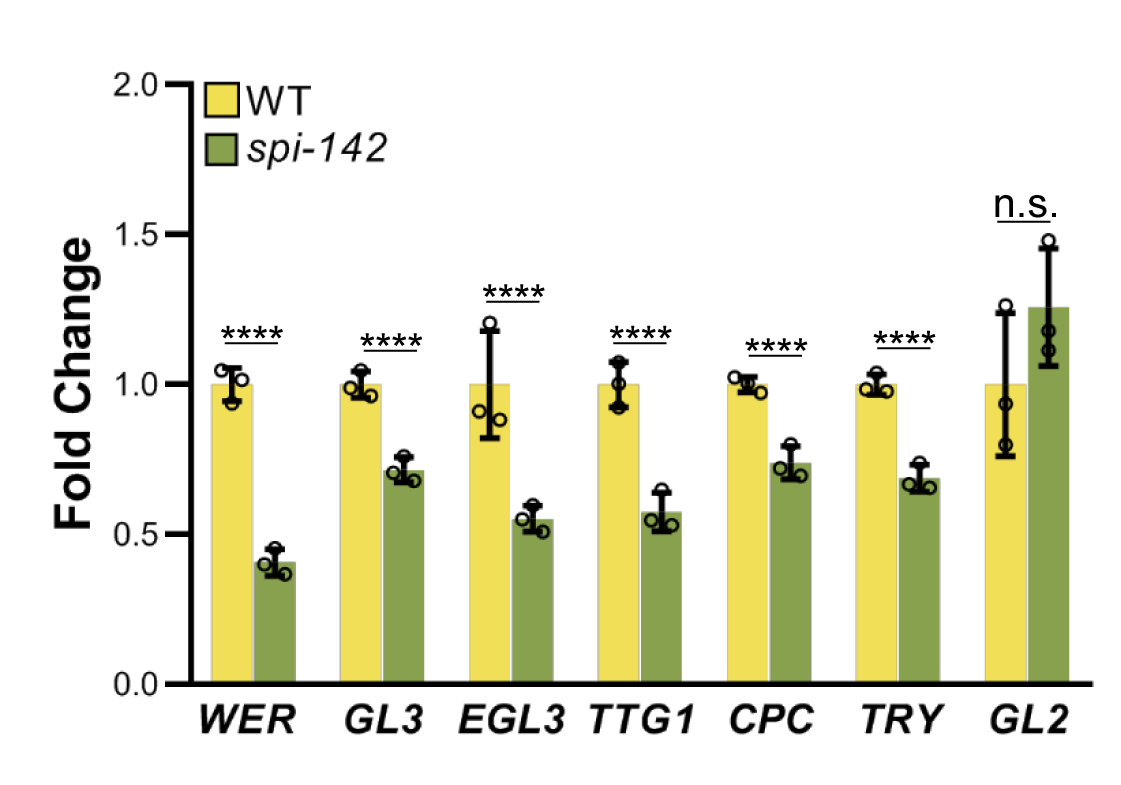

Supplement: Supplementary file 1 — Supplementary Material 1: Figure S1. Examination of expression levels of classical root hair genes in som1-1 mutant. Real-time quantitative RT-PCR (RT-qPCR) was used to evaluate the transcript levels of the target genes. Fold changes were calculated with respect to the expression levels in the WT. Data are mean ± SD of three biological replicates. ****P < 0.0001, n.s. not significant (P > 0.05, Student’s t test). [file 44154_2024_190_MOESM1_ESM.tif]

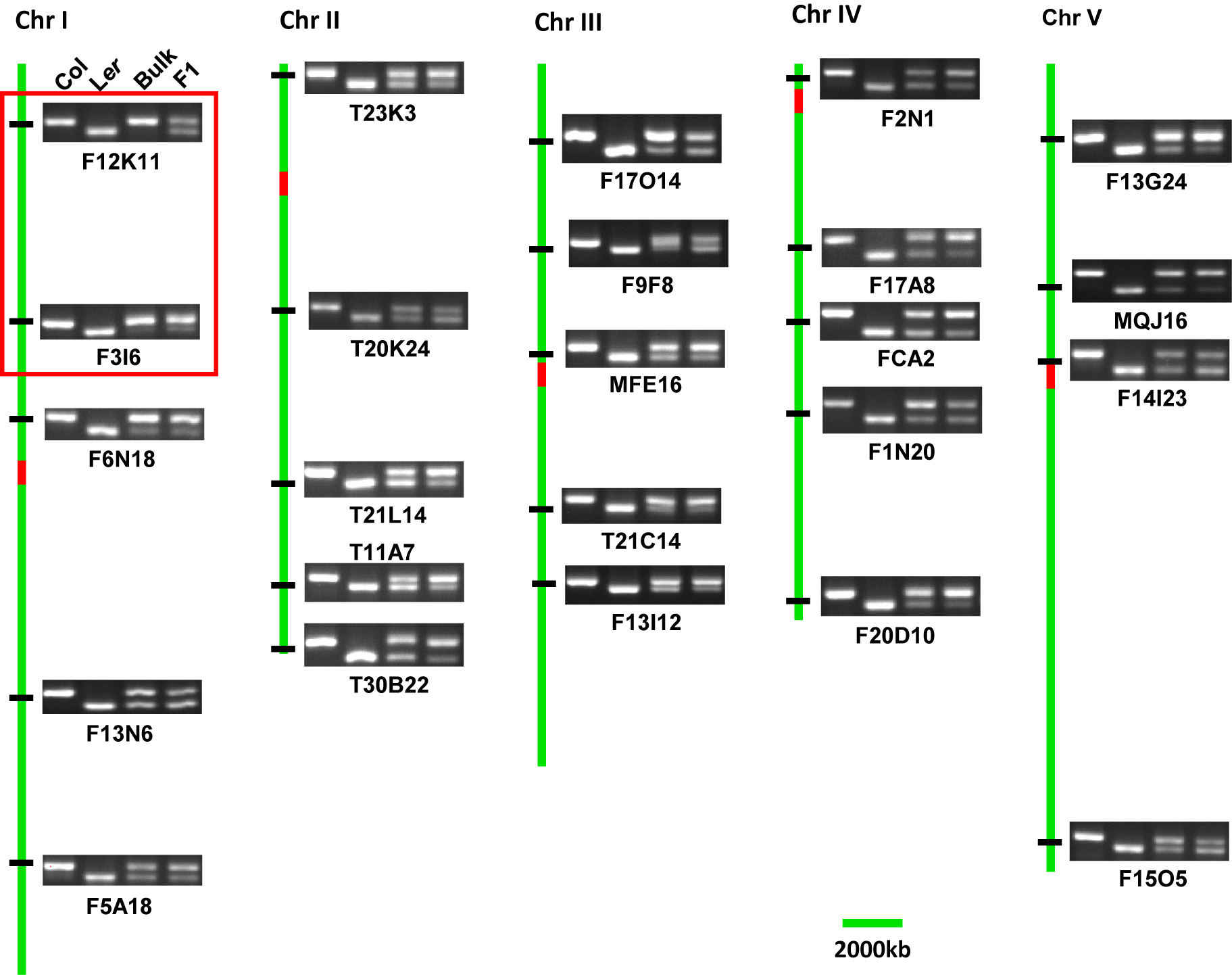

Supplement: Supplementary file 2 — Supplementary Material 2: Figure S2. Bulked segregant analyses (BSA) of SOM1. DNA pool were constructed by mixing the genomic DNA of 94 individuals from the F2 mapping population. SOM1 locus was localized near F12K11 and F3I6 on Chromosome I with 25 pairs of molecular markers that evenly distributes on the five chromosomes of Arabidopsis. Green lines represented the chromosomes and red bars indicated the centromeres. [file 44154_2024_190_MOESM2_ESM.tif]

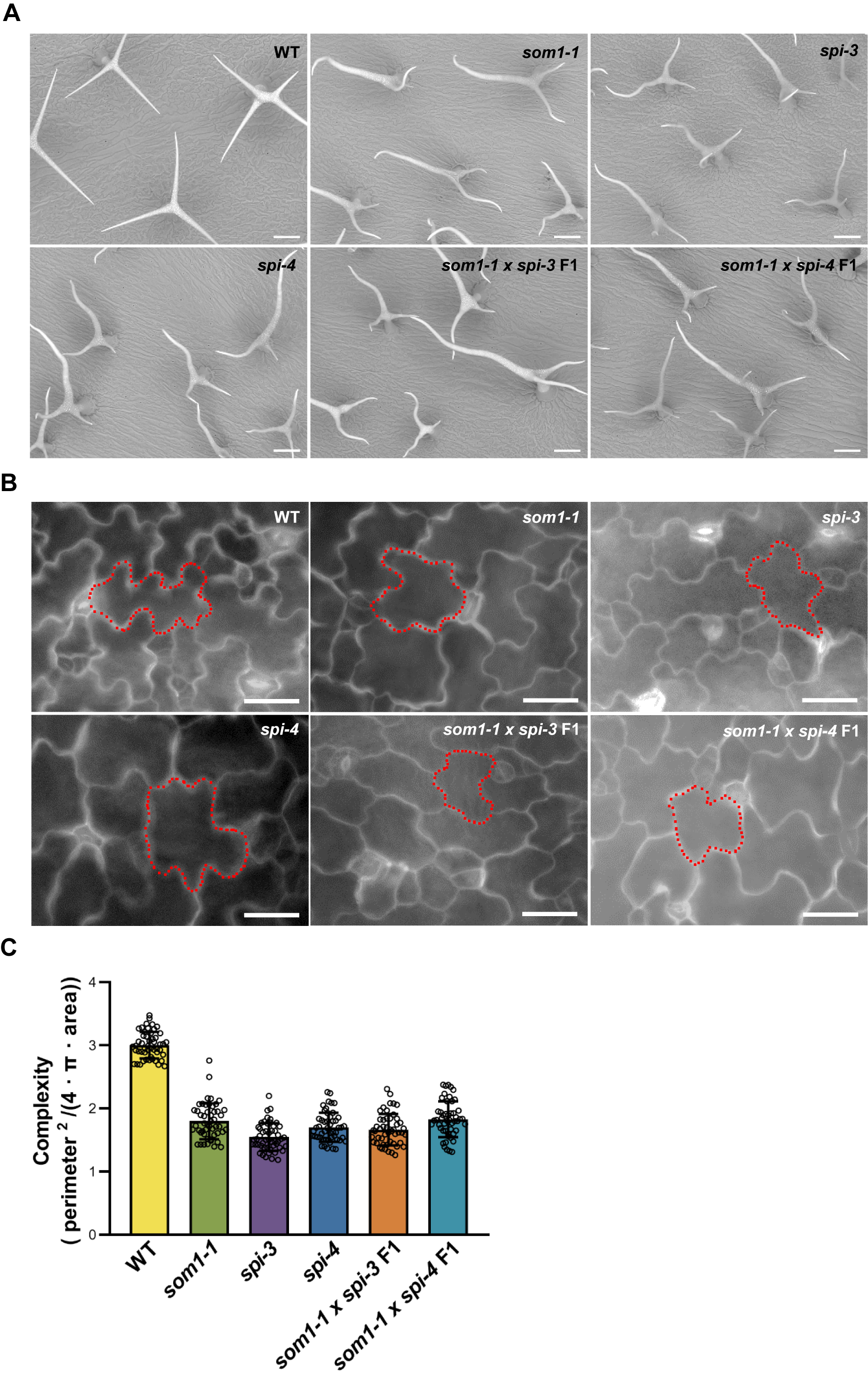

Supplement: Supplementary file 3 — Supplementary Material 3: Figure S3. Trichome branching and cotyledon pavement cell shapes in spi alleles. A Represented trichomes in the 5th rosette leaves of 2-week-old soil grown WT, spi mutants, and som1-1 × spi-3 F1as well as som1-1 × spi-4 F1 plants. Scale bars = 30 μm. B Cotyledon pavement cells of 4-day-old 1/2 MS medium grown plants indicated in A. Represented pavement cells were highlighted in red. Scale bars = 30 μm. C Quantification of complexity of cotyledon pavement cells in different genotypes show in A. Data are shown as mean ± SD (n = 50). [file 44154_2024_190_MOESM3_ESM.tif]

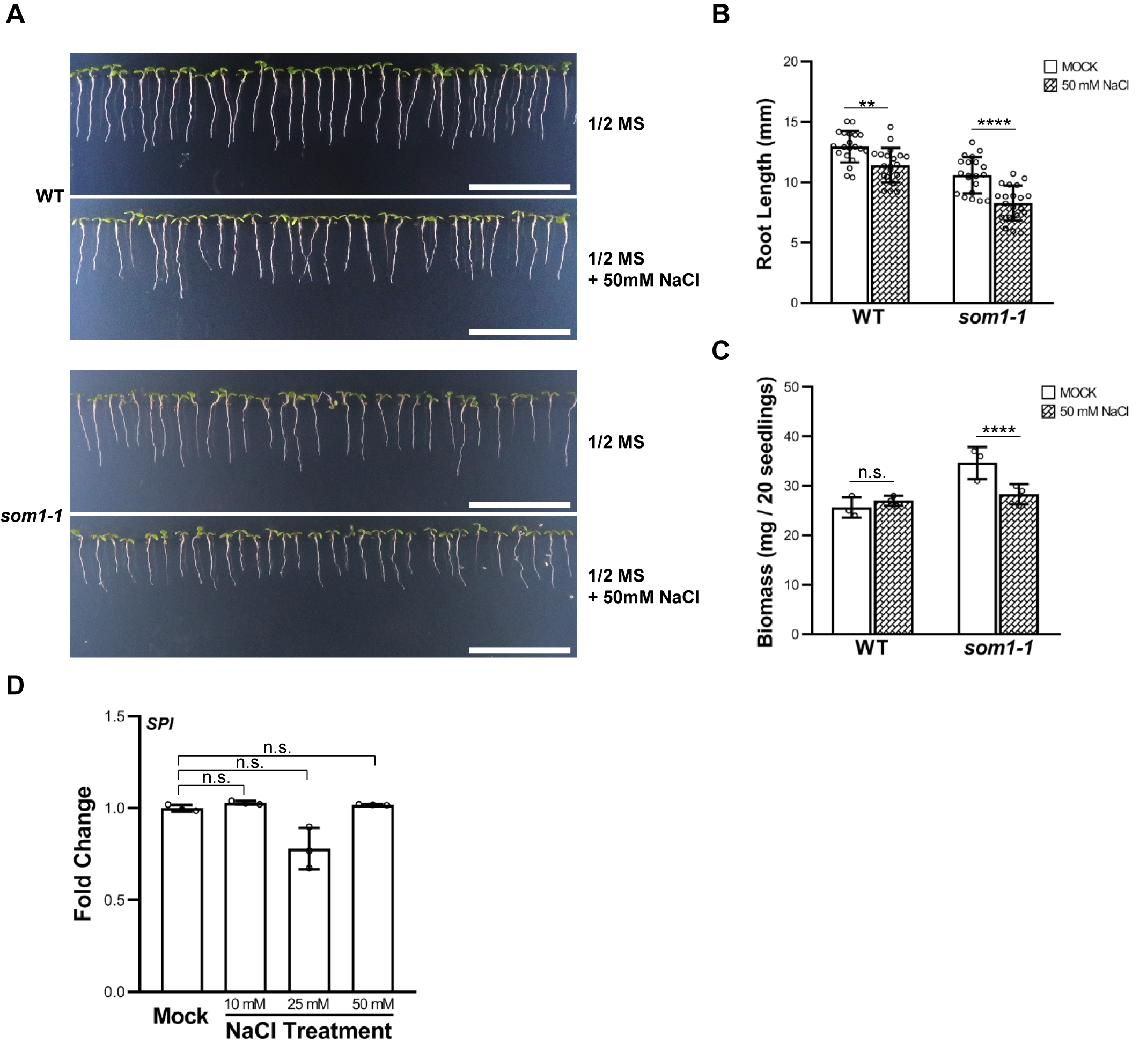

Supplement: Supplementary file 4 — Supplementary Material 4: Figure S4. Investigation of growth response of aerial parts and primary root of som1-1 to salt stress. A The grow status of 4-day-old WT and som1-1 seedling under salt stress. Scale bars = 2 cm. B and C Primary root length (B) and biomass (C) of 4-day-old WT and som1-1 seedling under NaCl treatment, respectively. D Transcription levels of SPI in various salt stress condition evaluated by RT-qPCR. For B, data are mean ± SD (n=20); for C and D, data are presented as mean ± SD of three biological replicates. **** P<0.0001, ** P<0.01, n.s. not significant (P > 0.05, Student’st test). [file 44154_2024_190_MOESM4_ESM.tif]

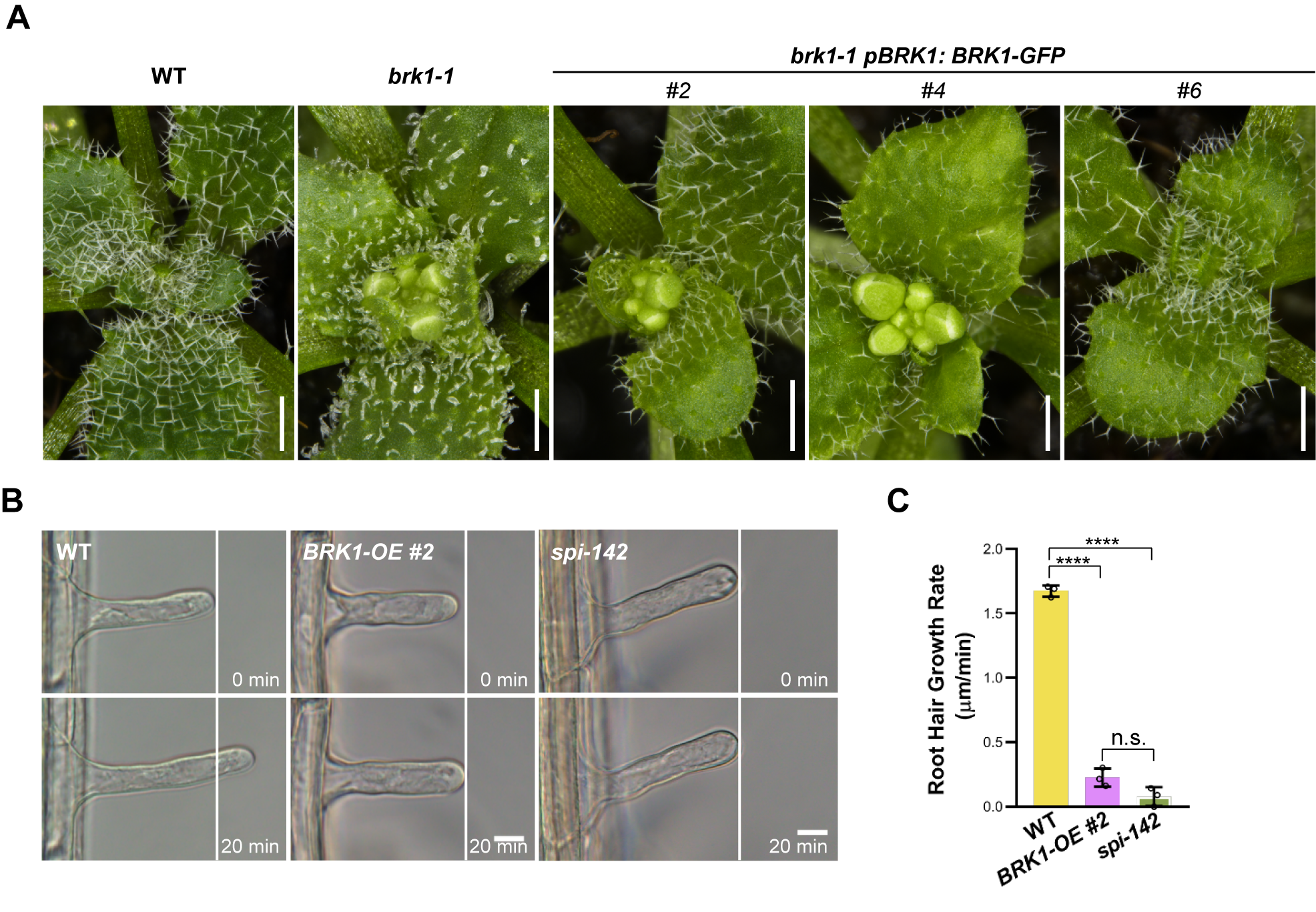

Supplement: Supplementary file 5 — Supplementary Material 5: Figure S5. Complemented analyses of brk1-1. A Trichome morphology in brk1-1 complemented lines. In planta expression of BRK1-GFP could restore the trichome defects of brk1-1. Scale bars = 1 cm. B Root hair growth curve of WT, BRK1 overexpression plants, and spi-142 mutant during a 20 min growth period. Scale bars = 25 μm. C Growth rate of the WT, BRK1 overexpression plants, and spi-142 root hairs. Data are presented as mean ± SD of three biological replicates. **** P<0.0001 (Student’s t test). [file 44154_2024_190_MOESM5_ESM.tif]

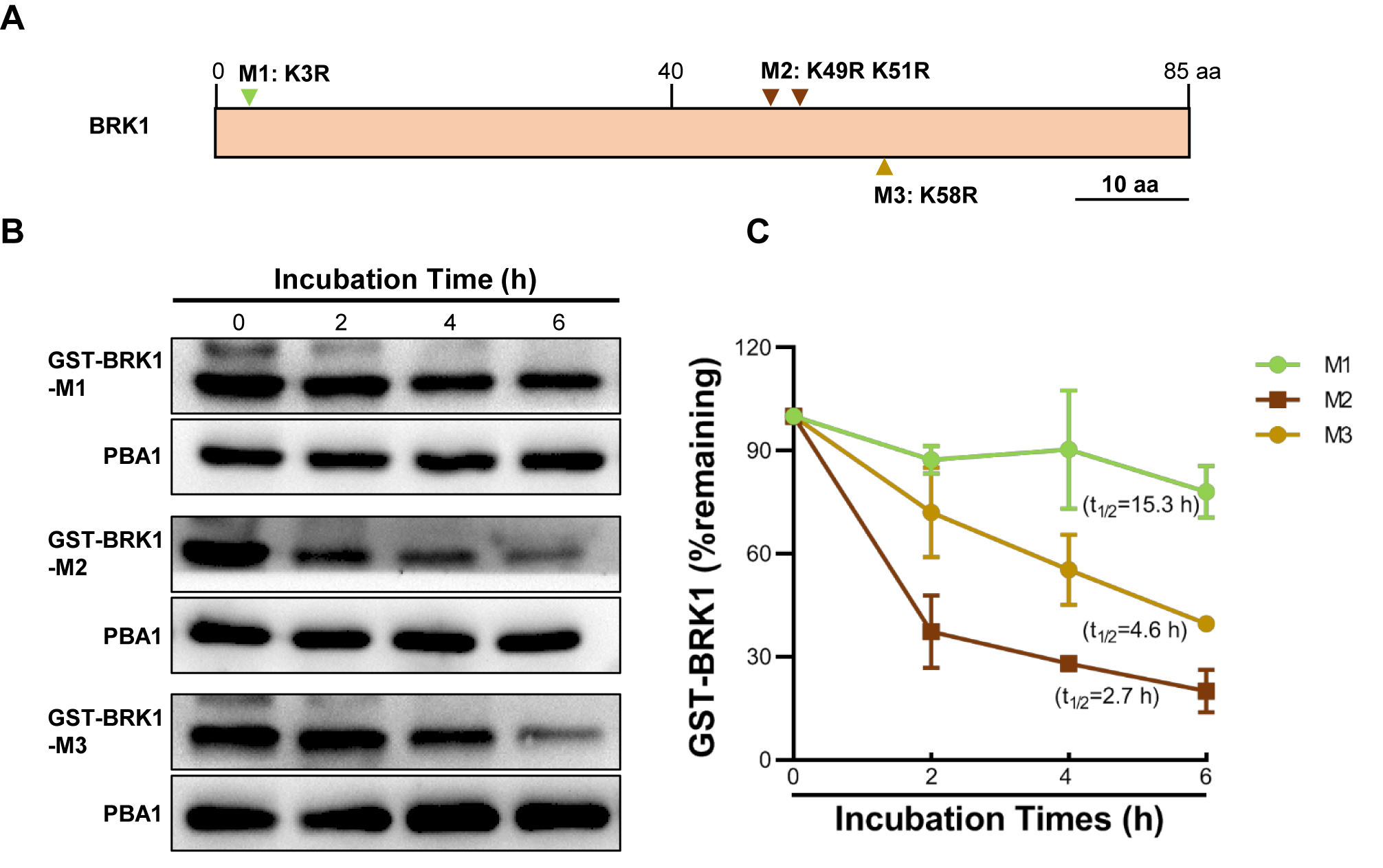

Supplement: Supplementary file 6 — Supplementary Material 6: Figure S6. Identification of lysine residues mediating BRK1 stability. A Schematic representation of the positions of mutated lysine residues in BRK1 protein. B In vitro cell-free protein degradation assay of mutated recombinant GST-BRK1s. Purified mutated recombinant proteins were added to cell extracts from WT plants and protein levels were examined at the indicated time by western blot. The amount of PBA1 was used as the loading control. C Half-life plots for cell-free degradation of mutated recombinant GST-BRK1s. The half-life times were predicted based on the regression equations. Data are means ± SD of three biological replicates. [file 44154_2024_190_MOESM6_ESM.tif]

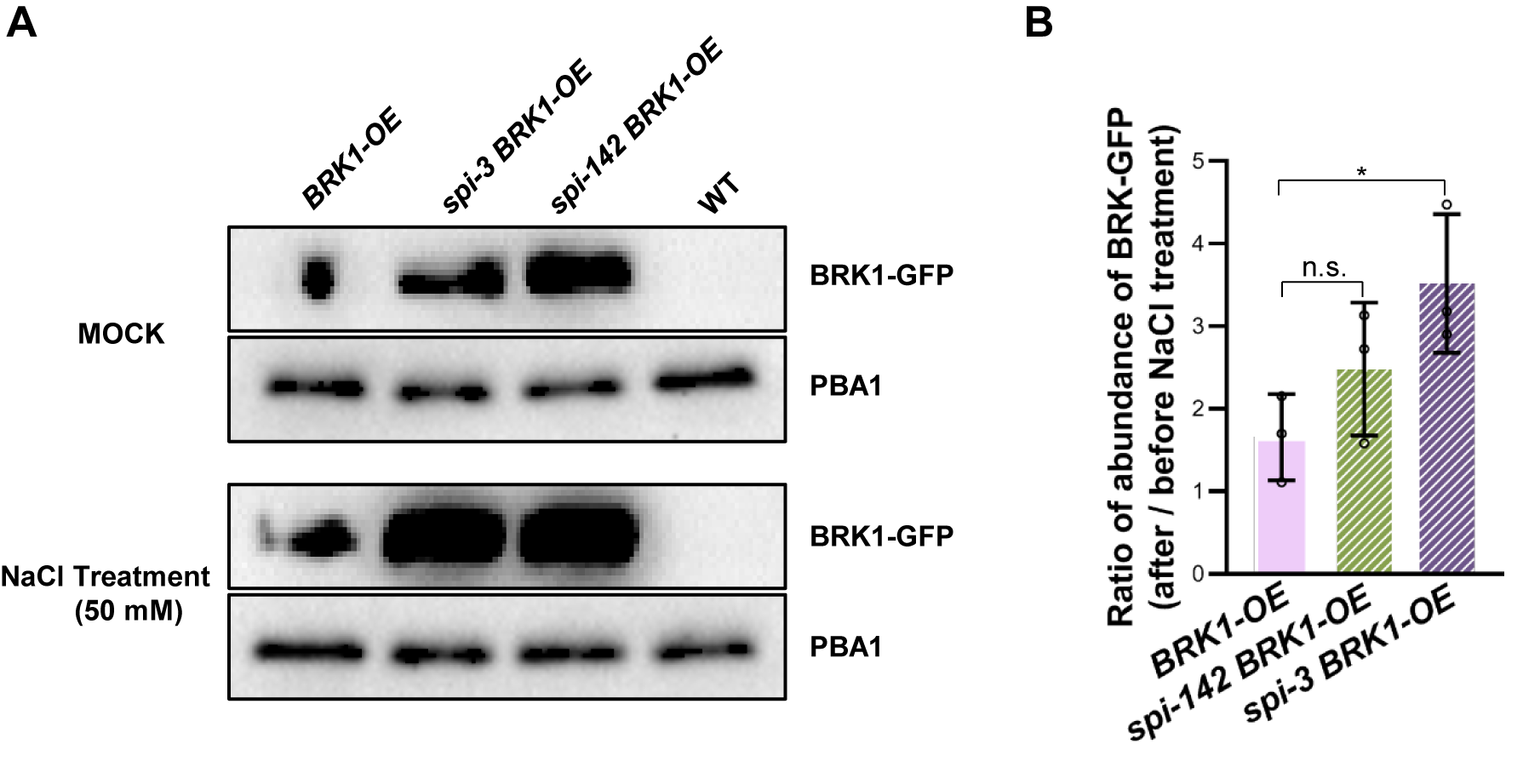

Supplement: Supplementary file 7 — Supplementary Material 7: Figure S7. Dissection the effects of salt stress on BRK1 stability. A Western blot analyses of BRK1-GFP abundance in 4-day-old pBRK1:BRK1-GFP, spi-142 pBRK1:BRK1-GFP, and spi-3 pBRK1:BRK1-GFP seedlings before and after NaCl treatment respectively with antibody anti-GFP. PBA1 levels were used as the loading control. B Quantitative analyses of BRK1-GFP abundance after NaCl treatment in plants with different genetic background. Data are means ± SD of three biological replicates. * P<0.05, n.s. not significant (P > 0.05, Student’s t test). [file 44154_2024_190_MOESM7_ESM.tif]
