# Supplementary material for: SPIRRIG is required for BRICK1 stability and salt stress induced root hair developmental plasticity in Arabidopsis
Source: Stress Biol. 2024 Nov 25;4(1):48. doi: 10.1007/s44154-024-00190-w (PMC11589064; doi:10.1007/s44154-024-00190-w)
Supplement: Supplementary file 8 — Supplementary Material 8: Table S1. The primer pairs used in this work. [file 44154_2024_190_MOESM8_ESM.docx]

**Table S1 The primer pairs used in this work**

| Name | Sequence (5´-3´) | Comments |
| --- | --- | --- |
| Salk_065311 LP  Salk_065311 RP | CCTGGTTCTATCTTCCTTGCC  CGTCATGATGGACCTAGTTGG | For homozygous mutant genotyping |
| GK_420D09 LP  GK_420D09 RP | TGGAGAACATTCTTCCACCAC  TGCTTGGGCAGAATATGATTC |  |
| CS86554 LP  CS86554 RP | TACAACCAATGAGCATACCG  GATCTCTCTTAGCACATGGA |  |
| F3I6 LP  F3I6 RP | TATTGGGCCT AGAAAAAGGC  TAATGTCATG TGTGGTCTGC | For positional cloning |
| F12K11 LP  F12K11 RP | ACATGGCCTTCTTATTCATTCT  AAGCTCATTCAACATTTGGC |  |
| T25N20 LP  T25N20 RP | CAGCACCTCTCCCTACTCCA  TTCGCGAGTACCTTCAGGAT |  |
| T1G11 LP  T1G11 RP | ATACCGAAGCTGAGCTGCAT  CACATTCTAAGCCTTGTAGAGATGA |  |
| F20D22 LP  F20D22 RP | CCACTAGCGAACCAGTGAGAAT  TGCAATCGTTGACCTTGGAG |  |
| som1-1 LP  som1-1 RP | TTGTCCTCAATATGGCTGGT  GGGTCGTCAATAAAAGAGAG | For *som1-1* mutant mutation site sequencing |
| BRK1 GST-LP  BRK1 GST-RP | CATGGATCCATGGCGAAAGCTGGAGGG  CATGTCGACTCACGTCGCAAACAGAGAAGG | For *pGEX4T-1-GST-BRK1* plasmid construction |
| BRK1-M1GST-LP | CATGGATCCATGGCGGCAGCTGGAGGG |  |
| BRK1-M1GST-RP | CATGTCGACTCACGTCGCAAACAGAGAAGG |  |
| BRK1-M2GST-LP | CAACCACAGCGAGCGCGTTGGCTTCTTTGAATGAG |  |
| BRK1-M2GST-RP | CTCATTCAAAGAAGCCAACGCGCTCGCTGTGGTTG |  |
| BRK1-M3GST-LP | TGAATGAGGCGTTGGATCTGTTGGAACG |  |
| BRK1-M3GST-RP | CGTTCCAACAGATCCAACGCCTCATTCA |  |
| BRK1 GFP-LP | CATGTCGACATGGCGAAAGCTGGAGGG | For *p35S:BRK1-GFP* plasmid construction |
| BRK1 GFP-RP | CATGGATCCCGTCGCAAACAGAGAAGGAT |  |
| pBRK1 LP | CATGTCGACGAGTAAGTGTGAGTGTAATCATG | For *pBRK1:BRK1-GFP* plasmid construction |
| BRK1 RP | CATTCTAGACGTCGCAAACAGAGAAG |  |
| BRK1 3'UTR F | CATGGATCCAACACCGTAGACGATATCG |  |
| BRK1 3'UTR R | CATGGATCCCTTTTTCGGTTTTTTCTTCCTCC |  |
| SPI (10)RT-LP | ATGGGGTGGCGATTATTCTTCC | For real-time quantitative RT-PCR |
| SPI (10)RT-RP | GGCCAGAAATACCAGTGACCAT |  |
| SPI (18)RT-LP | AACGTGATTACCAGCTGCGT |  |
| SPI (18)RT-RP | TCCTGAAATGGTTGTGTCGAGC |  |
| BRK1 RT-LP | GCAGATTGGGAGAATCGAGA |  |
| BRK1 RT-LP | TCACGTCGCAAACAGAGAAG |  |
| GAPDH RT-LP  GAPDH RT-RP | TTGGTGACAACAGGTCAAGCA  AAACTTGTCGCTCAATGCAATC | Internal control for real-time quantitative RT-PCR |
